# The ubiquitin conjugase Rad6 mediates ribosome pausing during oxidative stress

**DOI:** 10.1016/j.celrep.2023.113359

**Published:** 2023-11-02

**Authors:** Sezen Meydan, Géssica C. Barros, Vanessa Simões, Lana Harley, Blanche K. Cizubu, Nicholas R. Guydosh, Gustavo M. Silva

**Affiliations:** 1 National Institute of Diabetes and Digestive and Kidney Diseases, National Institutes of Health, Bethesda, MD 20892, USA; 2 Postdoctoral Research Associate Training Fellowship, National Institute of General Medical Sciences, National Institutes of Health, Bethesda, MD 20982, USA; 3 Department of Biology, Duke University, Durham, NC 27708, USA; 4 These authors contributed equally; 5 Lead contact

## Abstract

Oxidative stress causes K63-linked ubiquitination of ribosomes by the E2 ubiquitin conjugase Rad6. How Rad6-mediated ubiquitination of ribosomes affects translation, however, is unclear. We therefore perform Ribo-seq and Disome-seq in *Saccharomyces cerevisiae* and show that oxidative stress causes ribosome pausing at specific amino acid motifs, which also leads to ribosome collisions. However, these redox-pausing signatures are lost in the absence of Rad6 and do not depend on the ribosome-associated quality control (RQC) pathway. We also show that Rad6 is needed to inhibit overall translation in response to oxidative stress and that its deletion leads to increased expression of antioxidant genes. Finally, we observe that the lack of Rad6 leads to changes during translation that affect activation of the integrated stress response (ISR) pathway. Our results provide a high-resolution picture of the gene expression changes during oxidative stress and unravel an additional stress response pathway affecting translation elongation.

## In brief

Meydan et al. find a mechanism of translation control mediated by the ubiquitin conjugase Rad6. During oxidative stress, elongating ribosomes pause while making Ile-Pro peptide bonds, and the absence of Rad6 leads to non-canonical reprogramming of translation, implicating Rad6 as a regulator of the stress response.

## Graphical Abstract

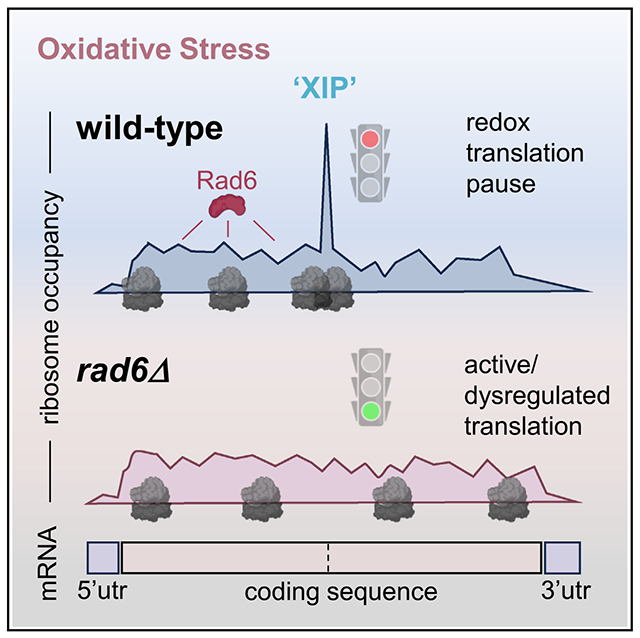

## INTRODUCTION

Eukaryotic organisms frequently encounter harmful environmental conditions, and this necessitates the timely and precise regulation of gene expression to support cellular stress defense, adaptation, and survival.^1^ Cellular stress caused by the accumulation of reactive oxygen species (ROS) affects important biomolecules such as nucleic acids, proteins, and lipids and is associated with several pathologies, such as cancer and cardiovascular and neurodegenerative diseases.^2–4^ ROS, including hydrogen peroxide (H_2_O_2_), can result from metabolic processes but also from exposure to a range of chemicals and environmental pollutants.^4,5^ Thus, oxidative stress occurs when ROS production overloads the cellular antioxidant defense. To prevent the detrimental effects of ROS, cells evoke an intricate regulatory network of gene expression at both the transcriptional and translational levels.^5–8^ Although the transcriptional response to oxidative stress has been more extensively studied,^6,9–12^ a genome-wide understanding of how cells regulate translation in response to oxidative stress is only beginning to be elucidated.^7,8,12–14^

In response to oxidative stress, eukaryotic cells reprogram translation by shutting down protein production globally while favoring the translation of essential proteins for cell survival.^5^ Some of these pathways are determined by the levels and availability of translation factors or are dictated by *cis*-regulatory mRNA sequences.^5,15^ Although an extensive number of studies have focused on the regulation of translation initiation,^8,16,17^ mechanisms of elongation regulation during stress are still not well understood.^18^

We previously discovered a new mechanism responsible for controlling translation elongation during oxidative stress via ubiquitination of ribosomes.^19–21^ This pathway is based on ubiquitin monomers linked by lysine 63 (K63), which mediates signaling functions independent of the proteasome.^20,22,23^ We named this pathway redox control of translation by ubiquitin (RTU).^24^ A key regulator of the RTU pathway is the E2 ubiquitin conjugase Rad6, which rapidly modifies ribosomal proteins with K63-linked polyubiquitin chains in response to H_2_O_2_.^19–21,25^ Ubiquitinated ribosomes arrest at the pretranslocation stage of translation elongation^21^; however, the mechanism by which ubiquitin traps ribosomes at this conformational stage is unknown. Furthermore, we recently showed that deletion of *RAD6* prevents K63-linked ubiquitination of ribosomes and leads to continued protein production under oxidative stress and dysregulated levels of antioxidant proteins.^25^ Rad6 is a multifunctional and highly conserved protein in which mutations to its human homolog UBE2A are associated with the X-linked intellectual disability type Nascimento.^26,27^ However, an understanding of the means by which Rad6 controls the translational landscape by modifying ribosomes and the crosstalk of the RTU pathway with other pathways of translation control remains elusive.

To characterize the translational landscape mediated by Rad6 under stress, we made use of next-generation sequencing of ribosome-protected mRNA fragments, also known as Ribo-seq or ribosome profiling,^28,29^ alongside RNA sequencing (RNA-seq). Moreover, we employed our recently improved Disome-seq approach that reveals ribosome collisions (disomes) and their connection to quality control and stress response pathways.^30^ Here, we found that upon hydrogen peroxide treatment, ribosomes from wild-type (WT) cells pause on isoleucine-proline sequences. Surprisingly, this redox-pausing signature was largely abolished upon deletion of *RAD6*. Furthermore, we showed that the RTU pathway functions independently of the ribosome-associated quality control (RQC) pathway, which is known for detecting and rescuing collided ribosomes. Finally, we showed that lack of Rad6 affects translation rates and activates additional translation programs, including the integrated stress response (ISR) through a non-canonical mechanism. Therefore, this study uncovers a critical mechanism of translational control and positions Rad6 as a key remodeler of the translation landscape through a ribosome-pausing mechanism.

## RESULTS

### Rad6 is required for redox pausing of ribosomes

**Figure 1.**
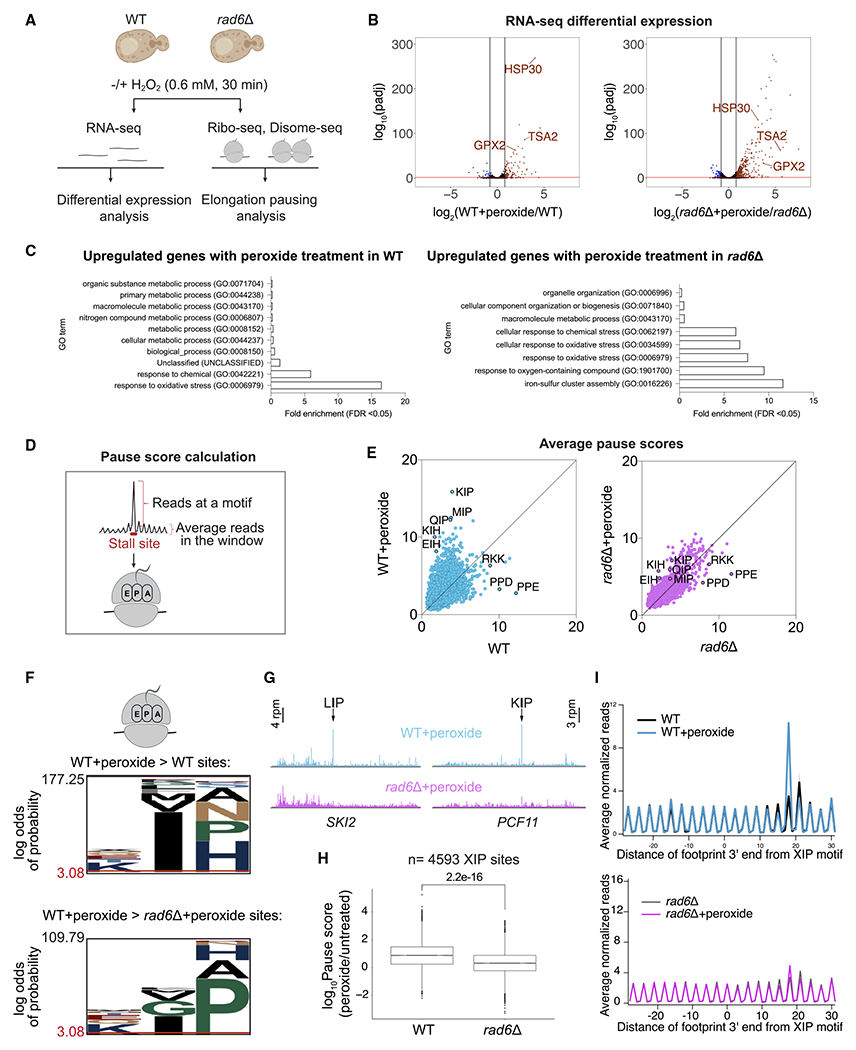
Rad6 is necessary for ribosome pausing during oxidative stress (A) Schematics of RNA-seq, Ribo-seq, and Disome-seq experiments conducted in WT and *rad6*Δ *S. cerevisiae* cells ± 0.6 mM H_2_O_2_ (peroxide) for 30 min. (B) Volcano plot showing differential RNA expression in WT+peroxide vs. WT (left) and *rad6*Δ+peroxide vs. *rad6*Δ (right). Genes that are significantly upregulated (log_2_fold change > 0.8, adjusted p value [padj] < 0.05) or downregulated (log_2_fold change < −0.8, padj < 0.05) as determined by DESeq2 analysis are shown in red and blue, respectively. The significance cutoff is indicated with a red bar. Known redox genes are labeled. (C) Significantly enriched Gene Ontology (GO) terms among genes upregulated upon peroxide treatment in WT and *rad6*Δ cells. GO analysis conducted in PANTHER, GO-Slim Biological Process, by using *S. cerevisiae* (all genes in database) as reference list and test type as FISHER with false discovery rate (FDR) correction. (D) Schematics for calculation of pause score, computed by dividing reads at a motif by the average reads in a window around the motif of interest (±50 nt). We calculated pause score at tri-amino acid motifs that map to the ribosomal E (penultimate position of the nascent peptide), P, and A sites. (E) Average pause scores of 6,267 tri-amino acid motifs plotted for untreated vs. peroxide data from WT (left) and *rad6*Δ (right) cells. Each point represents a tri-amino acid motif. Pause scores were calculated by applying a shift value of 18 nt from the 3′-end of the footprint, placing the first codon of the tri-amino acid motif in the E site. The average of two replicates is plotted. Prominent stalling motifs are labeled on the graph. Motifs that correspond to stalling that increases under peroxide are above the diagonal. (F) pLogo^32^ motif analysis of the tri-amino acid motifs that have 1.5-fold higher average pause score in WT+peroxide vs. WT samples (top, n_foreground_ = 1,004, n_background_ = 6,996) and in WT+peroxide vs. *rad6*Δ+peroxide (bottom, n_foreground_ = 397, n_background_ = 7,603) samples. The plots show enrichment for motifs with Ile in the P site and Pro in the A site for WT+peroxide samples vs. WT or *rad6*Δ+peroxide cells. (G) Example genes (*SKI2* and *PCF11*) with strong XIP redox pausing dependent on Rad6. The data are obtained from pooled biological duplicates. The stalling peaks corresponding to LIP and KIP motifs are indicated by arrows (top, blue traces) and are lost in the absence of Rad6 (bottom, pink traces). (H) Boxplot showing the significant loss of redox pausing at XIP sites (n = 4,593) in *rad6*Δ cells compared with WT. The significance of differences in the median of these pause score ratios was computed by independent two-group Mann-Whitney U test. (I) Average normalized Ribo-seq rpm mapped to genes aligned by their respective XIP motifs in WT (top) and *rad6*Δ (bottom) cells show loss of redox pausing at these motifs on average (top blue trace vs. bottom pink trace). Average of two replicates ± standard deviation (shaded) is plotted.

Rad6-mediated ubiquitination was suggested to affect translation during oxidative stress by arresting translation elongation at the pretranslocation stage.^19,21^ To further understand the impact of Rad6-mediated ubiquitination on ribosome pausing at a transcriptome-wide level, we conducted Ribo-seq experiments in WT and *rad6*Δ cells incubated with ±0.6 mM H_2_O_2_ (“peroxide” hereafter) for 30 min (Figures 1A and S1A).^28,31^ This peroxide concentration and the treatment time were optimized based on the peak accumulation of K63-linked polyubiquitin chains after the addition of peroxide to the media.^20^ Supporting the establishment of our system, RNA-seq experiments showed that the peroxide treatment resulted in significantly upregulated expression of genes involved in the oxidative stress response in both WT and *rad6*Δ cells (Figures 1B and 1C).

To understand the effect of Rad6 on translation elongation, we computed “average pause scores” for every possible combination of 3 amino acids positioned within the ribosome E, P, and A sites (Figure 1D; STAR Methods). First, we observed that amino acid sequences such as PPD, PPE, and RKK caused the strongest pausing in untreated WT cells (Figure 1E, left). However, the relative level of pausing at these sequences was not as high in stressed cells. Instead, we found that peroxide treatment in WT cells caused reprogramming of ribosome arrest and resulted in elevated pausing at specific sequence motifs, which we define as “redox pausing” (Figure 1E, left). These redox-pausing signatures were enriched in sites that have proline (Pro) and histidine (His) codons at the ribosomal A site. We also observed that stalling at isoleucine (Ile) codons at the ribosomal P sites increased upon peroxide treatment, especially in combination with A-site Pro codons (Figures 1E and 1F). Because we did not detect substantial enrichment for residues that specifically mapped to the E site (i.e., the amino acid corresponding to the penultimate, C-terminal position of the nascent peptide), we designated this stalling motif as “XIP,” where X refers to any amino acid.

Given prior evidence that Rad6-mediated ubiquitination could modulate translation elongation,^25^ we hypothesized that redox pausing would depend on Rad6. We therefore performed our pausing analysis in *rad6*Δ cells and found that redox-pausing signatures were strikingly lost (Figure 1E, right). The previously identified XIP redox-pausing motifs were the most susceptible to the loss of Rad6 (Figure 1F). Consistently, analysis of individual or averaged ribosome occupancy at XIP sites also revealed considerable loss of redox pausing in the absence of Rad6 (Figures 1G and 1H), where half of the XIP sites in the genome with redox pausing had decreased ribosome occupancy in *rad6*Δ cells (Figure S1B). In the untreated cells, ribosome stalling signatures looked similar between the two strains, except for increased stalling in *rad6*Δ cells at A-site Trp codons, a result that is specific to the SUB280 background used here (Figure S1C; see below for further discussion). Average ribosome occupancy at XIP motifs was consistent with the pause score analysis and revealed a peroxide-induced peak that was absent in untreated WT cells and in the *rad6*Δ strain (Figure 1I, top and bottom, respectively).

**Figure 2.**
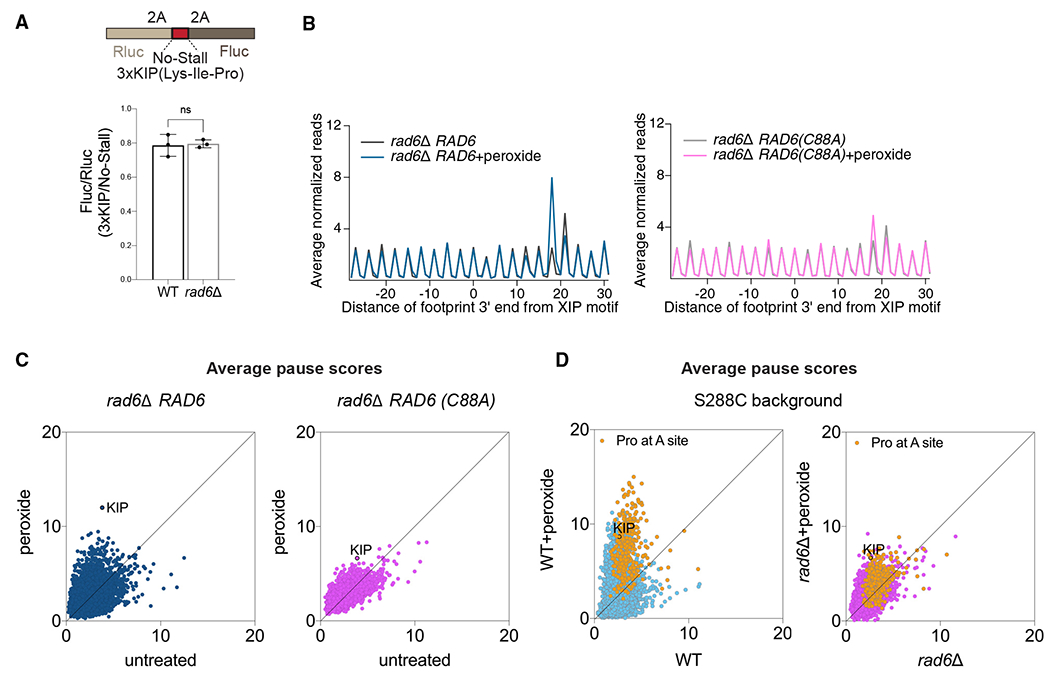
Ubiquitination activity of Rad6 mediates ribosome pausing but not rescue (A) Schematic for the Renilla-Firefly reporter construct used to measure ribosome rescue at a redox pausing motif (top, 3xKIP). The Fluc/Rluc ratio is expected to become lower when ribosomes dissociate from the mRNA (i.e., via ribosome rescue) after translating the Rluc sequence but prior to reaching the Fluc sequence. The ratio of the Fluc/Rluc value for the 3xKIP reporter compared with a no-stall reporter is shown (bottom). Note that the observed value of ~0.8 indicates some level of ribosome rescue due to the 3xKIP motif (value of 1.0 would indicate no rescue). Deletion of *RAD6* does not appear to affect ribosome rescue. The significance is assessed by unpaired t test. ns, not significant. Data from 3 replicates are shown. Error bar indicates mean ± standard deviation. (B) Average normalized Ribo-seq rpm mapped to genes aligned by their respective XIP motifs in *rad6*Δ cells complemented with either WT Rad6 (*rad6*Δ *RAD6*, left) or its catalytically dead mutant (*rad6*Δ *RAD6(C88A)*, right) show that ubiquitination activity of Rad6 is necessary to restore redox pausing at XIP. (C) Average pause scores of 6,267 tri-amino acid motifs plotted for untreated vs. peroxide data from *rad6*Δ cells complemented with *RAD6* (left) or *RAD6(C88A)* (right) show that expression of WT Rad6 restores overall redox pausing but the catalytically dead mutant does not. The KIP motif is labeled. (D) Average pause scores of 6,267 tri-amino acid motifs plotted for untreated vs. peroxide data from S288C WT and *rad6*Δ cells. Motifs with Pro codons at the A site are indicated in yellow and KIP motif labeled. These data indicate that the redox-pausing signatures and effect of Rad6 loss on them are consistent between different yeast strains.

Upon arresting at XIP motifs, ribosomes could resume translation or be rescued by quality control systems. To determine whether XIP motifs lead to ribosome rescue, we developed an inducible dual-luciferase reporter in which we can insert sequences between Renilla luciferase (Rluc) and Firefly luciferase (Fluc) coding regions that are expected to stall translation and lead to ribosome rescue. As a proof of principle, we determined that several sequences identified with a high pause score in our Ribo-seq data indeed impair the synthesis of Fluc (Figure S1D). Because of the global translation repression that occurs under oxidative stress (Figure S1E), this method did not allow us to measure dynamic changes in the Fluc/Rluc ratios during the short time window (30 min) following peroxide treatment used in this study. However, we still observed a reduction in the Fluc/Rluc ratio (Figures 2A and S1D; note that the Fluc/Rluc ratio equals 0.8 rather than 1.0) when 3xKIP was inserted in the absence of peroxide, consistent with an interpretation that there could be a loss of ribosomes between Fluc and Rluc in WT cells due to rescue or drop off. This trend was unchanged in *rad6*Δ cells, suggesting that Rad6 does not promote ribosome rescue.

Because oxidative stress induced by peroxide was previously associated with increased translation of 5′ and 3′ untranslated regions (UTRs),^7,13^ we tested whether Rad6 impacts translation outside of main open reading frames. Metagene analysis, performed by averaging data from genes aligned by their start or stop codons, showed modest changes in the occupancy of 5′ and 3′ UTRs with peroxide treatment in WT cells, as expected (Figures S1F–S1G). The absence of Rad6 did not affect these trends (Figures S1F–S1G), suggesting that Rad6 does not strongly impact translation of UTRs under oxidative stress.

To confirm our observation that Rad6 activity has a specific role in modulating redox pausing, we conducted additional Ribo-seq experiments in which we expressed both the WT Rad6 and the catalytically dead mutant Rad6 (Rad6^C88A^) in a *rad6*Δ background. Expression of WT Rad6 in *rad6*Δ cells restored peroxide-induced stalling at the XIP motif and other sites, whereas the ubiquitination-deficient mutant Rad6^C88A^ did not (Figures 2B and 2C). These results suggest that Rad6 catalytic activity is essential to regulate redox pausing.

To confirm the generality of our results, we also considered the effect of the yeast strain background. The yeast strains used in this study (SUB280 background) were constructed to express a single ubiquitin gene episomally.^33^ To test whether the unique properties of this strain were related to the observed redox-pausing signatures, we repeated experiments in the S288C background. As noted above, the *rad6*Δ cells in the S288C background lacked A-site pausing at Trp codons (Figure S1C), suggesting that this effect is not a feature of redox pausing. However, the S288C cells recapitulated the redox-pausing signatures at A-site Pro codons, including XIP motifs (Figure 2D). These data therefore show that redox pausing is a consistent mechanism of translational control in response to stress and that Rad6 plays a key role in this translation phenotype.

### Redox-pausing signatures are not mediated by the RQC pathway

We next investigated whether the well-established RQC pathway is involved in redox pausing. Stalled ribosomes can physically block upstream ribosomes from translating, resulting in the formation of a ribosome collision complex called a disome, where the two ribosomes interact.^34–36^ The RQC pathway is a cellular mechanism that detects disomes and promotes their removal from mRNAs.^37^ To evaluate whether peroxide treatment produced disomes, we performed Disome-seq^30,38,39^ in WT cells and found that the data exhibited redox-pausing signatures, such as XIP, and high dependence on Rad6 and generally mirrored our Ribo-seq data (Figures S2A–S2C). This suggests that some stalled ribosomes formed during oxidative stress collide with each other.

Previous studies showed that the E3 ubiquitin ligase, Hel2, triggers the RQC pathway by ubiquitinating collided ribosomes stalled at positively charged amino acid sequences, such as poly-Arg or poly-Lys.^34,40–43^ Ubiquitination leads to ribosome rescue but, in the absence of Hel2, collided ribosomes bypass these stall-inducing sequences and continue translating. Because the RQC pathway regulates translation arrest via Hel2-mediated ubiquitination of ribosomes, it raises the question of whether E3 Hel2 and E2 Rad6 cooperate in the same pathway of translational control.

**Figure 3.**
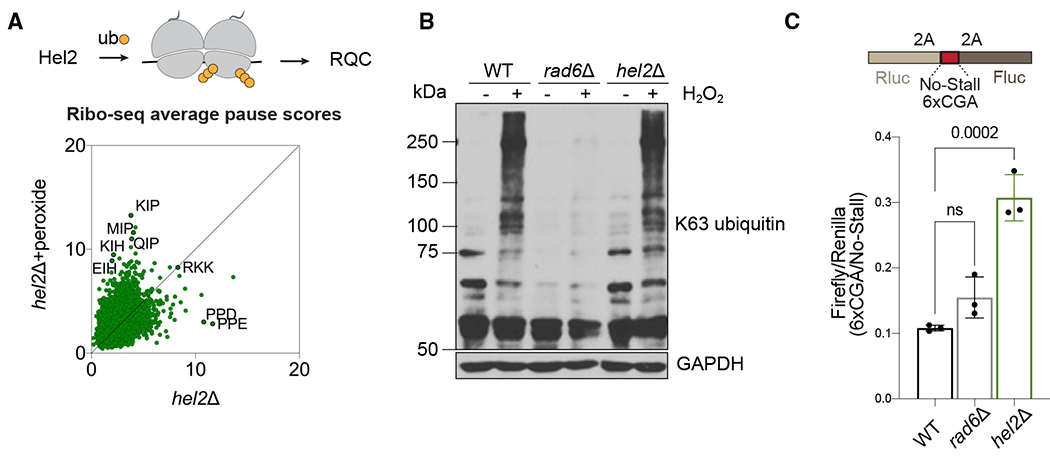
Redox pausing is not mediated by the RQC pathway (A) Hel2 is a E3 ligase that detects disomes and triggers the RQC pathway (top). Average pause scores of 6,267 tri-amino acid motifs plotted for untreated vs. peroxide data from *hel2*Δ cells show that redox-pausing signatures are intact in the absence of Hel2 (bottom). (B) Western blot demonstrates that deletion of *RAD6* eliminates peroxide-induced accumulation of K63-linked polyubiquitin chains, whereas *hel2*Δ does not. GAPDH is a loading control. Data are representative of two biological replicates. (C) The schematic of the Renilla-Firefly construct used to measure ribosome rescue of an RQC-targeting sequence (top, 6xCGA). The Fluc/Rluc ratio of the 6xCGA reporter compared with the no-stall reporter is shown (bottom). Deletion of *HEL2* causes increased Fluc/Rluc since ribosomes are no longer rescued, whereas deletion of *RAD6* does not significantly affect ribosome rescue at this sequence. The significance is assessed by one-way ANOVA test. ns, not significant. Data from 3 replicates are shown. Error bar indicates mean ± standard deviation.

To further investigate whether Hel2 is involved in rescuing disomes formed in response to stress, we performed a Ribo-seq experiment in *hel2*Δ cells. During peroxide treatment, XIP redox-pausing signatures were still present in cells lacking Hel2, which suggests a separation of functions (Figure 3A). Consistent with this, we also showed that loss of Hel2 did not affect the burst of K63-linked ubiquitination induced by peroxide treatment (Figure 3B). This finding further supports the notion that Rad6-mediated redox pausing is independent of Hel2 and the RQC. To explore the activity of Hel2 and Rad6 in rescuing stalled ribosomes, we inserted a known RQC-targeted stalling sequence consisting of 6 consecutive Arg codons (6xCGA) into our Rluc-Fluc reporter for ribosome rescue. This sequence is particularly problematic for the ribosome to translate due to I-C wobble codon-anticodon pairing.^44^ Ribosomes stalled at 6xCGA are known to be rescued by RQC,^44^ and as expected, we observed more ribosomes bypassing this stall-inducing site in the absence of Hel2 (Figures 3C and S2D). However, deletion of *RAD6* did not result in a significant increase in the Fluc/Rluc signal of the 6xCGA reporter (Figures 3C and S2D). These results suggest that Rad6 does not influence ribosome stalling and rescue in the same way as Hel2. We also used our 3xKIP reporter to check if ribosomes stalled by KIP sequences are targeted by Hel2 (Figure S2E). However, we did not observe an increase in the Fluc/Rluc signal of the 3xKIP reporter in the absence of Hel2, suggesting that ribosomes stalled at 3xKIP are not rescued by Hel2. Collectively, our findings indicate that Hel2 operates on a subpopulation of arrested ribosomes that likely does not include those (i.e., XIP motifs) that are enhanced by oxidative stress.

### Rad6 is required for translational repression during oxidative stress

**Figure 4.**
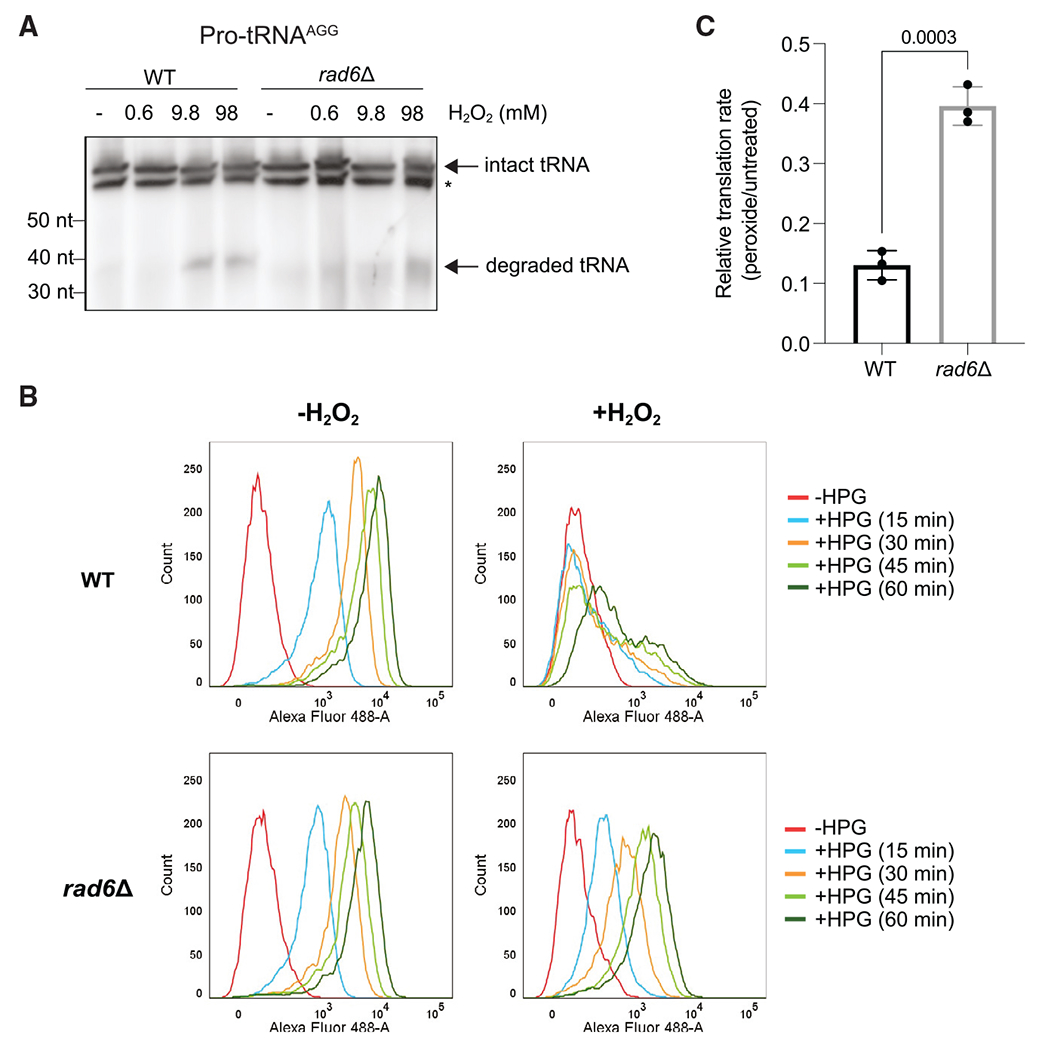
Rad6 promotes translation inhibition during oxidative stress (A) Northern blot by using a probe to Pro-tRNA^AGG^ shows that deletion of *RAD6* does not affect peroxide-induced degradation of this tRNA, which only takes place at very high concentrations of peroxide. The degradation fragment and intact tRNA are indicated by arrows. Asterisk (*) refers to a non-specific band. The peroxide-induced tRNA cleavage fragment of the WT cells is representative of two biological replicates, and two other replicates show that the intact tRNA levels do not change between WT and *rad6*Δ cells. (B) Flow cytometry histograms generated from the HPG incorporation assay showing the number of cells (y axis) and fluorescence magnitude (x axis) at indicated time points of HPG incubation (15–60 min). WT cells exhibit decreased HPG incorporation in the presence of peroxide (top). In contrast, HPG incorporation in *rad6*Δ cells is affected less by the peroxide treatment (bottom). (C) Quantification of HPG incorporation during peroxide treatment is shown as a normalized rate for HPG incorporation in treated vs. untreated cells. Translation rate in *rad6*Δ cells is less affected by peroxide than in WT cells. The translation rates were calculated by fitting the mean fluorescence values to a linear regression as a function of time. Significance was determined by two-tailed unpaired t test. Data from 3 replicates are shown. Error bar indicates mean ± standard deviation.

Having established that Hel2 and Rad6 affect ribosome stalling in different ways, we further explored mechanisms that could be responsible for redox-induced pausing. It was previously shown that peroxide treatment causes degradation of Pro-tRNA^AGG^, resulting in a ribosome stalled with an empty A site as it waits for binding of prolyl-tRNA.^45^ One possibility for the loss of redox pausing in *rad6*Δ cells could be that Rad6 indirectly or directly mediates the degradation of prolyl-tRNAs. Loss of Rad6 then might stabilize prolyl-tRNAs and thereby alleviate ribosome stalling at Pro codons. Using northern blotting for Pro-tRNA^AGG^, we found that the peroxide concentration that we used for our experiments (0.6 mM) did not result in tRNA degradation (Figure 4A, lane 2). Only at higher concentrations (9.8 mM, as used in Wu et al.,^45^ and 98 mM), were we able to detect the appearance of a tRNA fragment (Figure 4A, lanes 3–4, indicated by an arrow). We also observed that the magnitude and concentration dependence of prolyl-tRNA degradation induced by peroxide and the levels of intact prolyl-tRNA were similar in the *rad6*Δ strain (Figure 4A, lanes 5–8), further suggesting that loss of redox pausing in the absence of Rad6 is not due to changes in tRNA stability or levels.

We next assessed the overall rate of translation in *rad6*Δ cells to determine whether the absence of redox pausing affected the cell’s ability to produce proteins. We evaluated changes in translation rates by incorporation of a methionine analog, homopropargylglycine (HPG). This assay captures the totality of all effects on translation, including changes to both initiation and elongation, and provides an opportunity to evaluate mechanistic models. One possibility is that in the absence of Rad6, ribosomes would no longer undergo redox pausing and could therefore generate more protein during stress. Consistent with this model, the drop in the rate of translation measured by HPG incorporation in *rad6*Δ cells due to oxidative stress was significantly less than in WT cells (Figures 4B, 4C, and S3A). While translation in WT cells was severely inhibited by peroxide, cells lacking Rad6 were significantly less sensitive. In addition, the effect of Rad6 on peroxide-induced translational repression was reproducible in the S288C background (Figure S3B–S3C). These findings are also in agreement with previous data showing higher puromycin incorporation and GFP reporter expression in *rad6*Δ cells in the presence of peroxide compared with WT cells.^25^ Overall, our results suggest that Rad6-mediated redox pausing correlates with global repression of translation.

### Rad6 supports eIF2α phosphorylation under stress

**Figure 5.**
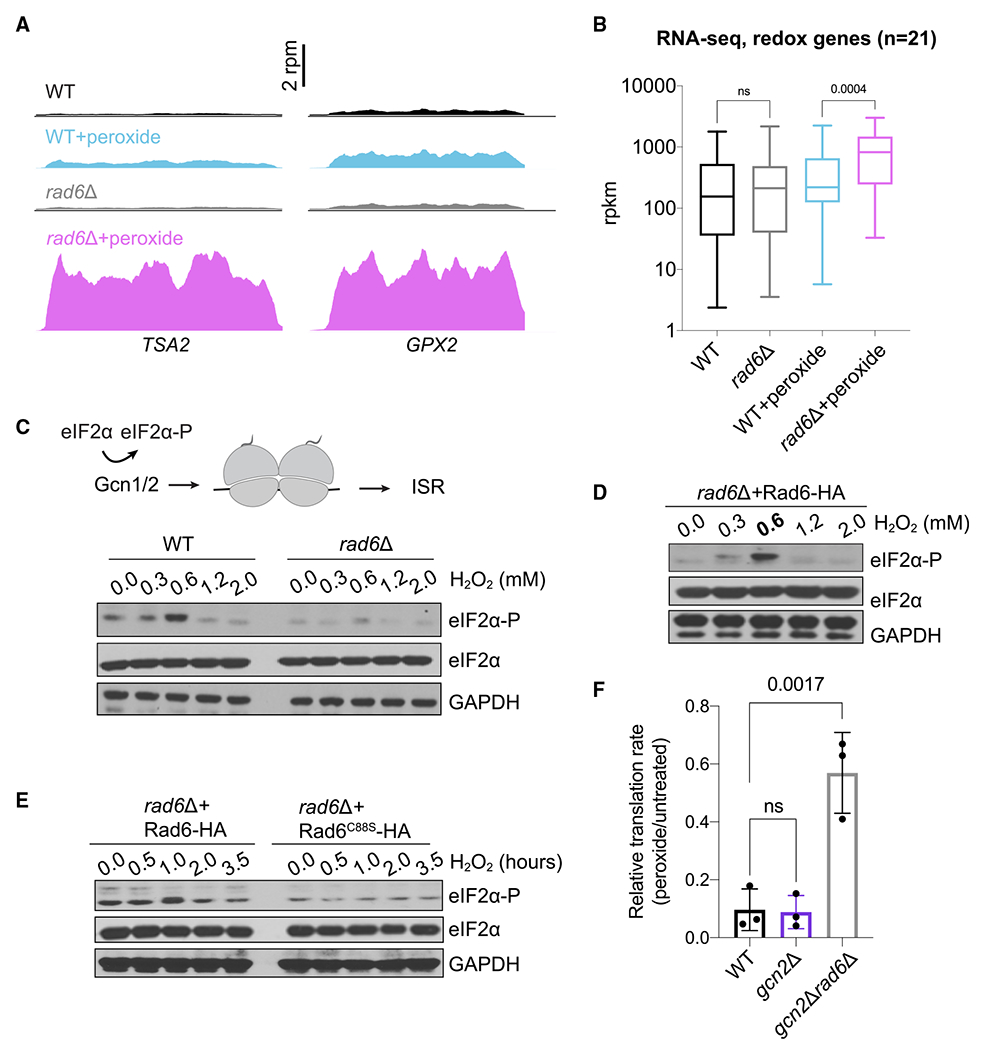
Rad6 promotes eIF2α phosphorylation (A) RNA-seq snapshots of *TSA2* and *GPX2* genes show that the lack of Rad6 causes increased expression of these mRNAs during peroxide treatment (pink vs. blue traces). Data are from pooled biological duplicates. (B) RNA-seq levels for the mRNAs encoding 21 redox enzymes show upregulation of these genes in *rad6*Δ cells under peroxide treatment. Significance is calculated by one-way ANOVA test. ns, not significant. Average data from 2 replicates were used to compute translation efficiency values. The line in the middle of each box is plotted at the median for the 21 genes, and whiskers show the minimum and maximum values. (C) Disome detection by Gcn1/2 induces eIF2α phosphorylation and ISR activation (top). Western blot demonstrates that eIF2α is phosphorylated upon oxidative stress, reaching a maximum at 0.6 mM peroxide, suggestive of increased ribosome stalling (bottom). Peroxide-induced eIF2α-P is reduced in the absence of Rad6, consistent with less ribosome stalling. eIF2α blots show that the total eIF2α levels do not change. GAPDH is used as a loading control.A biological replicate of these data at 0.6 mM peroxide is available in Figure S4E (lanes 1–4). (D) Western blot shows that the *rad6*Δ cells complemented with WT Rad6 (Rad6-HA) restore peroxide-induced eIF2α phosphorylation. eIF2α blots show that the total eIF2α levels do not change. GAPDH is used as a loading control. (E) Western blot shows that the *rad6*Δ cells complemented with WT Rad6 (Rad6-HA) restore peroxide-induced eIF2α phosphorylation but the catalytically dead mutant (Rad6^C88S^-HA) cannot, even at longer incubation times with 0.6 mM peroxide. eIF2α blots show that the total eIF2α levels do not change. GAPDH is used as a loading control. (F) Quantification of HPG incorporation during peroxide treatment is shown as the normalized rate for HPG incorporation in treated vs. untreated cells. The data show that the translation rate in *gcn2*Δ cells is affected by peroxide at a level similar to that in WT cells, but *gcn2*Δ*rad6*Δ cells are less affected by peroxide. The translation rates were calculated by fitting the mean fluorescence values to a linear regression as a function of time. Significance determined by one-way ANOVA test. ns, non-significant. Data from 3 replicates are shown. Error bar indicates mean ± standard deviation.

To understand the physiological impact of dysregulated translation in the absence of Rad6, we next explored by RNA-seq how peroxide affects the transcriptome in WT and *rad6*Δ cells. In the absence of oxidative stress (untreated), *rad6*Δ cells had significantly upregulated expression of multiple genes involved in metabolic processes (such as *GDB1*, *GPH1, PKP1*, and *DAK2*), the heat shock response (such as *HSP26, HSP30*, and *HSP78*), and the oxidative stress response (such as *GRX1*, *SOD2, TSA2*, and PRX1) compared with WT cells (Figure S4A). This suggests that even without oxidative stress, the lack of Rad6 causes a mild stress response. Upon peroxide treatment, we found that the expression of oxidative stress response genes is upregulated in *rad6*Δ cells beyond that found in the equivalently treated WT cells (Figures 1B, 5A, and S4B), consistent with the previous observation of increased ROS in *rad6*Δ cells under peroxide.^25^ When we limited our analysis to 21 genes coding for known antioxidant enzymes, we observed overactivated expression of these redox genes in *rad6*Δ cells in both untreated and peroxide-treated samples (Figures 5B and S4B). In addition, there were fewer ribosomal protein transcripts in *rad6*Δ cells, and this downregulation was exacerbated by peroxide treatment (Figure S4C). A decreased level of ribosomal protein transcripts is a hallmark of TOR inactivation, which is likely being driven by ROS in these cells.^5^ Although we did not identify an enrichment in XIP occurrence in redox genes, we observed that the translation efficiency of redox genes (ribosome footprints per transcript) was significantly increased in *rad6*Δ vs. WT cells under peroxide treatment (Figure S4D), which could be caused by faster initiation or slower elongation. Our previous work favors the former, as the production of several antioxidant proteins increases in *rad6*Δ cells under stress.^25^ These results support the model that in the absence of Rad6, increased ROS drives a distinct response at the RNA and translational levels.

Because *rad6*Δ cells seem to display a higher basal level of stress, we reasoned that loss of Rad6 could lead to the specific activation of the ISR pathway (also known as general amino acid control pathway in yeast), which is known to be induced by many stresses, including peroxide treatment.^5,15^ Oxidative-stress-induced ISR results in phosphorylation of eIF2α (phosphorylated eIF2α [eIF2α-P]) by the Gcn2 kinase and its coactivators Gcn1 and Gcn20.^46^ This leads to repression of overall translation while activating the transcription of stress response genes. In WT cells, we observed the expected induction of the ISR (increased eIF2α-P) during peroxide treatment, reaching its maximum level at 0.6 mM (Figure 5C). Surprisingly, however, phosphorylation of eIF2α in *rad6*α cells remained low in response to oxidative stress (Figures 5C and S4E; discussion). Expression of Rad6^WT^, but not its ubiquitination-deficient mutant (Rad6^C88S^), in *rad6*Δ cells restored eIF2α-P at 0.6 mM peroxide (Figures 5D and 5E). Even longer incubation times with peroxide did not result in increased levels of eIF2α-P in *rad6*Δ cells expressing the mutant Rad6^C88S^ (Figure 5E). These observations could be explained by a model where the lack of Rad6 affects the expression of the ISR machinery and thereby impairs regulation of eIF2α phosphorylation. To test this possibility, we checked whether the components of the ISR machinery, Gcn1, Gcn2, and Gcn20, are properly expressed in the absence of Rad6. The Ribo-seq and RNA-seq data showed no difference in RNA levels and translation efficiency (TE) of the genes coding for Gcn1/2/20 proteins (Figure S4F), suggesting that the absence of Rad6 does not directly affect the expression of ISR machinery components. Another possibility is that loss of Rad6 activates the TOR pathway, which in turn inhibits Gcn2 activity.^47,48^ However, our RNA-seq data showed that the TOR pathway is likely inhibited in *rad6*Δ cells (Figure S4C), suggesting that loss of ISR activation in *rad6*Δ cells is not due to TOR activation. Taken together, these data showed that the activity of Rad6 promotes eIF2α phosphorylation during oxidative stress, though the mechanism underlying this effect remains unclear.

Because Rad6 promotes peroxide-induced eIF2α phosphorylation, we next asked whether Rad6-mediated translation repression during oxidative stress (Figure 4) is due to the inhibition of translation initiation (caused by eIF2α-P) or the inhibition of translation elongation (consequence of Rad6-mediated redox pausing). Our HPG incorporation experiments in *gcn2*Δ cells showed similar levels of peroxide-induced translation repression to WT cells (Figure 5F). However, translation was derepressed in *gcn2*Δ*rad6*Δ cells (Figure 5F), suggesting that the translation elongation block induced by Rad6 plays a major role in peroxide-induced translation inhibition.

### Lack of Rad6 induces *GCN4* translation

**Figure 6.**
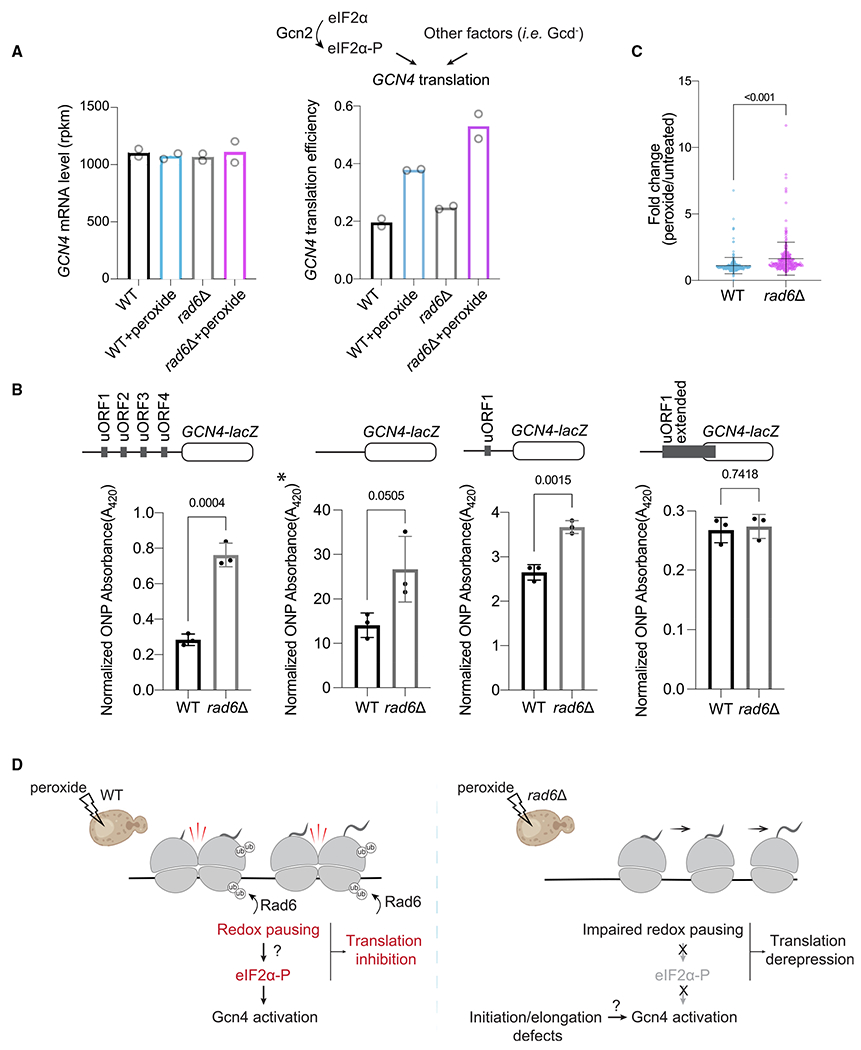
Lack of Rad6 induces translation of *GCN4* (A) eIF2α phosphorylation or other events induce translation of *GCN4*. Bar chart shows that the mean *GCN4* TE (Ribo-seq reads normalized to RNA-seq reads for *GCN4* main ORF, right graph) increases with peroxide in both WT and *rad6*Δ cells, while *GCN4* RNA-seq levels remain constant (left graph). TE of *GCN4* is higher in *rad6*Δ cells. (B) Reporter assay for *GCN4* activation. *GCN4* activation is assayed by *lacZ*, which is fused to the *GCN4* coding sequence. At the top, constructs used are shown: all 4 native uORFs (left), without all uORFs (mid-left, *GCN4* constitutive translation), with only uORF1 (mid-right, control for activation), and an extended version of uORF1 (right, to assess leaky scanning). The y axes show ONPG absorption values at 420 nm, normalized by total protein levels. *Due to saturation, the experiment was performed with 1/10 total protein, and the extrapolated values are shown for comparison. The statistical significance was calculated by unpaired t test. The data show that loss of Rad6 increases *GCN4* translation and that this effect is not due to leaky scanning. Data from 3 replicates are shown. Error bar indicates mean ± standard deviation. (C) Peroxide-induced expression of Gcn4’s transcriptional targets (n = 250, see STAR Methods for further details) assessed by RNA-seq shows that constitutive translation of *GCN4* in *rad6*Δ cells also leads to increased expression of its downstream genes. Average of two replicates used to make the plot. The statistical significance was calculated by unpaired t test. Line indicates mean, and bars indicate standard deviation for the 250 genes. (D) Model for the oxidative stress response. In WT cells (left), oxidative stress causes ubiquitination of ribosomes by Rad6 to persist, which leads to redox pausing and collisions. Collisions trigger eIF2α phosphorylation, which leads to translation of *GCN4* and transcription of its target mRNAs. Both increased ribosome pausing and phosphorylation of eIF2α could contribute to inhibition of global translation in WT cells. In *rad6*Δ cells (right), redox pausing is impaired due to lack of ubiquitination, and this results in lower eIF2α phosphorylation. Both impaired redox pausing and lower eIF2α-P could contribute to translation derepression. Despite the lack of peroxide-induced eIF2α phosphorylation, Gcn4 is still activated in *rad6*Δ cells.

Because we observed reduced peroxide-induced eIF2α-P in *rad6*Δ cells (Figure 5C), we expected that the ISR, and its associated effects on the translation of the *GCN4* gene, would be minimal. However, while the transcript level remained constant (Figure 6A, left graph), we noticed that translation of the *GCN4* gene was increased in the *rad6*Δ vs. WT strain, and this was true both in the presence and in the absence of peroxide (Figure 6A, right graph). This is an unexpected effect because the accumulation of eIF2α-P is the typical driver of increased translation of the *GCN4* mRNA, which encodes a transcription factor that upregulates many ISR genes.^49^

We therefore explored mechanisms that could affect the translational control of *GCN4*. The *GCN4* gene has 4 upstream open reading frames (uORFs). After translating uORF1 or uORF2, many ribosomes are not fully recycled, and the 40S subunits remain on the mRNA.^49,50^ These 40S subunits then resume scanning and rebind the eIF2-containing ternary complex (TC) (eIF2-GTP-tRNA_i_-Met), which allows them to reinitiate translation, typically at uORF3 or uORF4. Termination and recycling after these uORFs are generally efficient, which prevents the ribosomes from reinitiating again and translating the *GCN4* main ORF. During stress conditions, eIF2α-P reduces TC levels and thereby increases the odds that 40S subunits bypass uORF3 and uORF4 and instead reinitiate translation at the *GCN4* main ORF. In addition, leaky scanning, where ribosomes skip the uORFs and instead initiate at the downstream *GCN4* main ORF, or other initiation defects can also lead to increased *GCN4* translation. Because eIF2α is minimally phosphorylated in *rad6*Δ cells during oxidative stress (Figure 5C), the observed high TE of *GCN4* could be either due to initiation defects or leaky scanning. To monitor *GCN4* main ORF expression in relationship to its uORFs, we used a *GCN4-lacZ* reporter assay, which includes the natural context of *GCN4* with all 4 uORFs (Figure 6B). Consistent with Ribo-seq and RNA-seq experiments (Figure 6A), the *GCN4-lacZ* reporter showed that *rad6*Δ cells have higher Gcn4 levels compared with WT cells in the absence of stress (Figure 6B, left bar chart). As a positive control for the reporter, we treated the cells with 3-amino-1,2,4-triazole (3-AT), which mimics amino acid starvation by inhibiting His biosynthesis. 3-AT increases ribosome stalling at His codons and thereby activates Gcn2, leading to phosphorylation of eIF2α and higher *GCN4* translation.^51^ Expectedly, the *GCN4-lacZ* reporter showed increased expression upon 3-AT treatment in both WT and *rad6*Δ but not *gcn2*Δ cells (Figure S5A).

We next examined variations of the *GCN4-lacZ* reporter where all uORFs or uORFs 2–4 had been removed (Figure 6B, middle). We found that the higher expression level in *rad6*Δ vs. WT cells that we observed for the reporter with all uORFs intact was maintained or slightly less in both of these reporters. These data therefore suggest that the derepression of *GCN4* in *rad6*Δ cells relies on a mechanism that is mostly independent of the uORFs. This conclusion is consistent with the observation that the expression of genes known to modulate *GCN4* expression in a uORF-dependent way (“Gcd genes”)^49^ did not change in our RNA-seq and Ribo-seq data (Figure S5B). To test whether leaky scanning plays a role in the activation of *GCN4* translation in *rad6*Δ cells, we used another reporter where uORF1 is repositioned downstream and extended to overlap with the beginning of the *GCN4* main ORF. The only way scanning ribosomes could reach the *GCN4* main ORF would be if they scanned past uORF1 via leaky scanning. The preferential expression of *GCN4-lacZ* we observed in *rad6*Δ vs. WT cells was not maintained in the uORF1-extended reporter (Figure 6B, right bar chart), showing that the lack of Rad6 does not increase leaky scanning. Together, the above results establish that Rad6 loss can lead to increased Gcn4 protein in the cell without an increase in eIF2α phosphorylation and offer important insights on the mechanism.

Finally, our data suggested this activation of *GCN4* translation in the absence of eIF2α-P may also be true in the presence of peroxide. We showed that *rad6*Δ cells that are peroxide treated had a lower level of eIF2α-P compared with similarly treated WT cells (Figure 5C). However, the translational efficiency of *GCN4* was higher in these peroxide-treated *rad6*Δ cells (Figure 6A, right graph, pink bar higher than blue bar), and this was also true for the *GCN4-lacZ* reporter (Figure S5C, gray bar with peroxide higher than black bar with peroxide). Moreover, RNA-seq data showed that known Gcn4-target mRNAs^52^ were upregulated at the transcriptional level in *rad6*Δ cells upon peroxide treatment compared with WT cells (Figure 6C). This indicates that the production of Gcn4 without eIF2α-P leads to the expected functional outcome. Our results therefore suggest a model where loss of ubiquitination by Rad6 causes dysregulation of eIF2α phosphorylation but that constitutive translation of *GCN4* in *rad6*Δ cells offers compensation (Figure 6D).

## DISCUSSION

Our work revealed an oxidative stress response pathway that regulates translation through the E2 ubiquitin conjugase Rad6. We found that Rad6 plays a key role in redox pausing of ribosomes as well as the expression of Gcn4, all contributing to the maintenance of cellular homeostasis during oxidative stress.

Although inhibition of translation initiation by eIF2α phosphorylation plays a key role in repressing translation during oxidative stress, previous data showed that even in the absence of Gcn2 (the sole eIF2α kinase in yeast), translation continues to be inhibited.^8^ We previously showed that Rad6-mediated K63-linked polyubiquitin chains change the conformation of the ribosome, and this conformation could block translation elongation.^21^ Consistent with this idea, we found here that oxidative stress leads to pausing at specific sequence motifs, particularly XIP, and that this was dependent on Rad6 (Figures 1E–1I and 2B–2D) and its ubiquitin conjugation activity (Figures 2B and 2C). Strikingly, Rad6 was necessary for a substantial portion of translational repression that is induced by oxidative stress (Figures 4B, 4C, S3, and 5F), further implicating redox pausing in the inhibition of protein synthesis. We did not observe significant functional enrichment or changes in TE for genes with strong redox-pausing scores, which suggests that XIP acts globally to control translation. Interestingly, TE goes up for a set of antioxidant genes in the absence of Rad6, which hints that redox transcripts may be particularly affected (Figure S4D). Therefore, like many stress pathways such as the ISR, the RTU pathway likely includes global and specific outcomes.

Although the mechanisms of redox pausing are not entirely clear, we ruled out changes to tRNA stability^45,53^ by showing that the peroxide concentration used herein does not lead to Pro-tRNA^AGG^ degradation (Figure 4A). However, oxidative stress is known to affect tRNA modifications,^54^ and these changes can influence ribosome pausing.^55^ Therefore, it is possible that altered modification of other tRNAs could contribute to P-site pausing signatures, such as Ile, in our data. These pausing events could be exacerbated when combined with a poor peptidyl-transfer substrate at the A site, such as Pro. Interestingly, Pro acts as a scavenger of ROS,^56–59^ and therefore Pro amino acid levels (and tRNA aminoacylation) may also be affected by oxidative stress. In *S. pombe*, peroxide was shown to cause ribosome pausing and collisions at Trp codons due to decreased levels of charged Trp-tRNA.^14^ It is therefore possible that lower levels of Pro-tRNA charging could contribute to XIP pausing.

One model for Rad6-mediated ubiquitination is that it ensures the efficiency of a general step in the elongation cycle so that loss of Rad6 would lead to slower elongation. The peaks on the redox-pausing motifs (i.e., XIP) would decrease, as a new step in the cycle would become rate limiting. However, this seems unlikely since the overall translation rate is not reduced in *rad6*Δ cells under oxidative stress (Figures 4B, 4C, and S3). Alternatively, Rad6-mediated ubiquitination could cause ribosomes to stall during oxidative stress (i.e., at XIP motifs), and loss of Rad6 would eliminate these events. Our findings support this model (Figure 6D) since translation is consistently higher in peroxide-treated *rad6*Δ cells compared with WT (Figures 4B, 4C, and S3). Rad6 could specifically target stalled ribosomes, making them longer lived and easily detectable. Rad6-induced stalling is consistent with the RTU pathway inhibiting translation by slowing elongation during oxidative stress (Figures 4 and 5F). Although we observed ribosome collisions at XIP motifs by Disome-seq (Figure S2), the collisions are likely not the sole mechanism for translation inhibition, as individually stalled ribosomes also contribute to slowed elongation.^18^

Even in the absence of oxidative stress, K63-linked ubiquitin supports polysome stability^19,60^ and accumulates upon deletion of the deubiquitinating enzyme encoded by *UBP2*.^20^ Because Rad6 is also constitutively associated with polysomes,^25^ our data suggest that basal levels of ribosome ubiquitination are important to control the translation cycle, which provides an explanation for how the deletion of *RAD6* could reduce translation rate even in non-stress conditions (Figures 4B and S3).

Another point that remains unclear is the fate of ubiquitinated ribosomes upon stress cessation. One possibility is that they are rescued, perhaps by proteins in the RQC pathway. However, our rescue-based reporter experiments showed that lack of Rad6 did not affect the Fluc/Rluc ratio in the reporter containing the redox-pausing motif XIP (Figure 2A), suggesting that ubiquitination of ribosomes by Rad6 does not lead to rescue. This finding is also consistent with reports of K63-ubiquitinated ribosomes being in polysomes and likely engaged in translation.^20^ The other, and more plausible, scenario is that ubiquitin marks are removed from the ribosome once the oxidative stress insult is no longer present. We favor this possibility because the deubiquitinase Ubp2 removes K63 ubiquitin on the ribosome and the activity of this enzyme is mediated by peroxide levels in the cell.^20^ In agreement with this model, K63-linked ubiquitin modification has been shown to be necessary for the stability of polysomes.^19,60^

Although the lack of Hel2 did not affect redox pausing and K63-linked ubiquitination (Figures 3A and 3B), it is possible that oxidative stress induced by other ROSs could lead to ribosome collisions that are targeted by RQC.^61,62^ Therefore, different ROSs and their modes of production, abundance, target, and subcellular location could potentially engage unique pathways of translation control mediated by the Hel2 and Rad6 pathways.

In addition to RQC, we also examined the relationship of Rad6 with the ISR. Peroxide is known to trigger the ISR, a pathway that is activated by ribosome collisions^30,63^ and leads to eIF2α phosphorylation. Therefore, our observation that Rad6-mediated stalling (and ribosome collisions) is induced by peroxide suggests one way that oxidative stress could lead to eIF2α phosphorylation. The loss of redox pausing in the absence of Rad6 is consistent with the lack of eIF2α phosphorylation. While eIF2α-P can inhibit translation initiation during cellular stress, under the conditions of our experiment, inhibition is mostly driven by the elongation block induced by Rad6 (Figures 4 and 5F). Interestingly, although the effect of Rad6 on redox pausing was reproducible in both SUB280 and S288C strains, we observed that impaired induction of eIF2α phosphorylation by peroxide was stronger in the SUB280 strain (Figure S4E). This suggests that other cellular inputs or differing disome levels may contribute to eIF2α phosphorylation in the S288C strain. Consistent with this idea, it has been reported that *GCN4* translation is not activated by peroxide in the S288C strain^5^ and that S288C has a unique genetic background that affects mitochondria physiology and cellular redox biology,^64^ which could influence redox experiments.

Despite impaired eIF2α phosphorylation, *GCN4* is still constitutively translated (Figures 6A and 6B) in *rad6*Δ cells, even in the absence of oxidative stress, and this effect appears to be somewhat independent of the uORFs (Figure 6B). Therefore, other changes in translation likely drive the constitutive translation of *GCN4*. One possibility is that we previously observed increased subunits of eIF3 in polysomes from WT cells compared to cells unable to produce K63-linked ubiquitin chains,^19^ suggesting that the level of polysome recruitment or ubiquitination of translation factors could affect translation in cells lacking Rad6. Alternatively, prior work suggested that changes in mRNA levels, due to loss of decapping, leads to increased *GCN4* translation and phosphorylation of eIF2α during stress. Interestingly, this occurred without global inhibition of translation, potentially due to the altered levels of capped mRNAs.^65^ As we observed Rad6-dependent changes in mRNA levels (Figures 1B, 5B, and S4A–S4C), the role of differentially expressed mRNAs may also be important for the effects of Rad6 on *GCN4* translation. Future studies will be necessary to determine how Rad6 integrates translation and transcription.^66,67^

Mutations in the human homolog of Rad6, UBE2A, are linked to intellectual disability type Nascimento due to loss of UBE2A activity.^27,68^ UBE2A was also shown to modulate neuronal function in flies by interacting with the E3 ubiquitin ligase Parkin and thereby inducing mitophagy.^69^ We previously showed that UBE2A complements Rad6 function in the RTU pathway and that Rad6 carrying the corresponding disease mutations leads to dysregulated K63-linked polyubiquitination response during oxidative stress in yeast,^25^ further supporting the idea that the role of Rad6 in homeostasis is conserved. Our studies in yeast, therefore, reveal crucial insights into the cellular response to UBE2A deficiency and could be important for delineating the disease mechanisms.

## LIMITATIONS OF THE STUDY

A limitation of the study is that we still lack mechanistic details on how Rad6 mediates this selective XIP ribosome pausing under stress and how these pauses impact the overall reprogramming of protein synthesis in response stress. Our prior data support a model where ubiquitination of ribosomal proteins by Rad6 is required to stabilize elongating ribosome at these positions. Finally, because of the multiple functions of Rad6, future work is needed to understand their distinct contributions to regulate gene expression under stress.

## STAR★METHODS

### RESOURCE AVAILABILITY

#### Lead contact

Further information and requests for resources and reagents should be directed to and will be fulfilled by the lead contact, Gustavo Silva (gustavo.silva@duke.edu).

#### Materials availability

Plasmids and strains generated in this paper are available upon request and completion of a Material Transfer Agreement.

#### Data and code availability

- High-throughput sequencing data are available on NIH GEO archive GSE226082. Raw (uncropped) images for western blots and source data for all plots are available online at Mendeley Data. When replicates were performed, they are described in the figure legends and source data files. Source data and replicates for gel images that are not included in the manuscript are available on Mendeley Data: https://doi.org/10.17632/j99gggz7ys.1.
- Custom scripts created in this paper have been deposited at Github and the accession link is provided in the Key Resource Table.
- Any additional information required to reanalyze the data reported in this work paper is available from the lead contact upon request.

### EXPERIMENTAL MODEL AND STUDY PARTICIPANT DETAILS

The yeast strains used in this study are listed in Table S1. SUB280 strain derivatives were grown in synthetic defined (SD) medium composed of D-Glucose (BD Difco, #215510), yeast nitrogen base (BD Difco, #291940) and drop-out amino acid medium without Leu and Trp (Sigma, #Y0750). SUB280 *rad6*Δ *RAD6* and SUB280 *rad6*Δ *RAD6(C88A)* cells were grown in SD media supplemented with drop-out amino acid supplements without Leu, Trp and Ura (Sigma, #Y1771). S288C strain derivatives were grown in SD complete media by using drop-out amino acid supplements without Leu and Trp (Sigma, #Y0750) and supplementing it back with L-leucine (Sigma, #L800) and Tryptophan (Sigma, #T8941). Starter cultures were grown at 30°C overnight and then diluted to an OD_600_ of 0.001 (*rad6*Δ cells) or 0.0001 (WT cells) and were grown to a final OD_600_ between 0.5 and 0.6 for ~16 h. Unless noted otherwise, the cultures were treated with freshly diluted H_2_O_2_ (peroxide) (Sigma, #216763), achieving a final concentration of 0.6 mM, for 30 min, filtered and frozen in liquid nitrogen for Ribo-seq, Disome-seq and RNA-seq experiments. Unless stated otherwise, data shown in the paper are produced from the yeast strain SUB280.

### METHOD DETAILS

#### Ribo-seq, disome-seq and RNA-seq experiments

Ribo-seq, Disome-seq and RNA-seq experiments were performed based on published protocols.^30,31,39^ Frozen yeast cell pellets and frozen droplets of lysis buffer (20 mM Tris pH 8.0, 140 mM KCl, 1.5 mM MgCl_2_, 1% Triton X-100 and 0.1 mg/mL cycloheximide [Sigma, #C7698]) were lysed using a Retsch Cryomill (Retsch 20.749.0001). The resulting powder of frozen cell and lysis buffer mixture was thawed at room temperature, transferred to a 50 mL falcon tube to spin at 3000 g for 5 min at 4°C. The supernatant was then spun at 21000 g for 10 min at 4°C. The absorbance of the supernatant (cell lysate) at 260 nm was recorded and total “OD” of the lysate was calculated as the product of the volume (in mL) multipled with A_260_ reading. A fraction of the lysate equivalent to OD = 45 was flash frozen in liquid nitrogen. Prior to RNase I digestion, lysates were thawed, diluted with an equal volume of lysis buffer and then digested with ~26 U of RNase I (Ambion, #AM2294) per OD for 1 h at room temperature (22°C) with gentle agitation at 700 rpm. Monosome (for Ribo-seq) and disome (for Disome-seq) fractions were separated by loading the lysates onto a 10%–50% sucrose gradient, prepared in gradient buffer (final concentration: 20 mM Tris pH 8.0, 150 mM KCl, 5 mM MgCl_2_, 0.5 mM DTT), and spun at 40,000 rpm for 3 h at 4°C using an SW 41 Ti Swinging-Bucket Rotor (Beckman Coulter). Sucrose gradient fractionation was performed by using a Brandel Density Gradient Fractionation System. The peaks corresponding to monosomes and disomes were collected and RNA was purified by using the SDS, hot acid phenol-chloroform extraction method. For RNA-seq, the total RNA was isolated directly from the frozen cell pellets by the SDS, hot acid phenol-chloroform extraction method and fragmented in a buffer (pH 9.2) containing 12 mM Na_2_CO_3_, 88 mM NaHCO_3_, 2 mM EDTA for 35 min at 95°C. The total RNA was cleaned up using the Oligo Clean & Concentrator kit (Zymo Research, #D4060). Monosome/disome footprints and total RNA isolated as described above were run on a 15% TBE-Urea polyacrylamide gel (Bio-Rad, #3450091) for the size selection process. For Ribo-seq, Disome-seq and RNA-seq, RNA fragments between 25 and 34 nt, 54–68 nt and 50–70 nt were excised from the gel, respectively. We used the 50 nt band from a small RNA marker (Abnova, #R0007) for RNA-seq experiments and other RNA size markers used for size selection are listed in Table S2. The excised gel pieces were frozen on dry ice for 30 min and thawed in RNA extraction buffer (0.3 M NaOAc, 1 mM EDTA, 0.25% SDS) overnight at 20°C with gentle agitation (700 rpm). Next day, RNA was precipitated and the pellet was resuspended in 10 mM Tris pH 8.

#### Next generation sequencing library preparation

Library preparation was conducted by following published protocol.^31^ The RNA fragments from Ribo-seq, Disome-seq and RNA-seq experiments were first dephosphorylated using PNK (NEB, #M0201L) and ligated to preadenylated linkers containing a 5 nt-long random Unique Molecular Index (‘UMI’) and a 5 nt barcode that is unique for each sample (listed in Table S2). The linkers that were pre-adenylated using a 5′ DNA adenylation mix (NEB, #E2610L) were ligated to dephosphorylated RNAs using T4 truncated RNA ligase 2 (K227Q) (NEB, #M0351L). Unligated linkers were depleted by using 5 U per sample of 5′ deadenylase (NEB, #M0331S) and RecJ exonuclease (Biosearch Technologies, #RJ411250). Ligated RNA samples with unique barcodes were pooled and cleaned up using the Oligo Clean & Concentrator kit (Zymo Research, #D4060). All samples were next reverse transcribed by using Superscript III (Invitrogen; 18080044), and the reverse transcription primer (NI-802, listed in Table S2) containing a random 2 nt UMI. At this step, ribosomal RNA (rRNA) was removed from Disome-seq and RNA-seq samples by using Qiagen FastSelect (Qiagen, #334215). The cDNAs obtained from this reaction were resolved on a 10% TBE-Urea gel (Bio-Rad, #3450089) and cDNAs were extracted using DNA gel extraction buffer (0.3 M NaCl, 1 mM EDTA, 10 mM Tris pH 8) with gentle agitation (700 rpm) overnight at 20°C. The next day, DNA was precipitated and the pellet was resuspended in 10 mM Tris pH 8. The footprints were circularized using CircLigase ssDNA Ligase (Biosearch Technologies, #CL4115K). For Ribo-seq samples, rRNA removal was performed at this stage by oligonucleotide substraction using Dynabeads MyOne Streptavidin C1 (Invitrogen, #65001) and DNA oligos that are the reverse complement of ribosomal RNAs (listed in Table S2). The samples were then amplified by PCR using Phusion DNA Polymerase (ThermoFisher Scientific, #F530L) and resulting product were resolved in hand-poured 8% native TBE gel. The libraries were extracted using DNA gel extraction buffer (0.3 M NaCl, 1 mM EDTA, 10 mM Tris pH 8) with gentle agitation (700 rpm) overnight at 20°C. The next day, DNA was precipitated and the pellet was resuspended in 10 mM Tris pH 8 to obtain the final library. For Disome-seq of *rad6*Δ cells, four different PCR libraries were pooled to increase the yield due to lower levels of disome population in these cells. Quality of the library was assessed by using a BioAnalyzer via the High Sensitivity DNA Kit (Agilent, #5067-4626) and TapeStation via High Sensitivity D100 Screen Tape System (Agilent, #5067–5584, #5067–5585). Sequencing experiments were performed by the NIDDK Genomics Core and NHLBI DNA Sequencing and Genomics Core at NIH (Bethesda, MD). Sequencing of SM099F, SM100F, SM103F-SM110F samples was conducted on an Illumina HiSeq2500 machine (single end, 50 bp cycle) and the rest of the samples on an Illumina NovaSeq machine (single end, 100 bp cycle).

#### Computational processing and analysis of Ribo-seq, disome-seq and RNA-seq data

The sequencing data was processed as described previously.^30^ Custom scripts are available on Github (https://github.com/guydoshlab). Briefly, fastq files of sequencing samples were provided by NIDDK Genomics Core and NHLBI DNA Sequencing and Genomics Core (NIH). We used Cutadapt^71^ to remove linkers and demultiplex for retrieving individual samples from pooled data. For RNA-seq samples, we trimmed all the reads to 50 nt by using following parameters: -j 0 -L 57 –discard-untrimmed. To remove rRNA and tRNA reads, we then aligned the files to an index of noncoding RNAs with Bowtie version 1.1.2^70^ by using following parameters: -v 2 -y -S -p 12. We removed PCR duplicates by using a custom python script. We then aligned the deduplicated files to coding regions and splice junctions of R64-1-1 S288C reference genome assembly (SacCer3, Saccharomyces Genome Database Project) by using the following parameters: -v 1 -y -a -m 1 –best –strata -S -p 4. The number of reads that were obtained after each of these steps are outlined in Table S3.

Custom python scripts are used for the data analysis by using biopython version 1.72 and python 2.7.18. For Ribo-seq and Disome-seq experiments, only the reads between 25-34 and 57–63 nt were analyzed, respectively. Ribo-seq and Disome-seq reads were aligned by their 3′ ends. For RNA-seq experiments, 50 nt reads were analyzed and coverage of reads was used instead of 3′ alignment. All reads were normalized in units of rpm (reads per million mapped reads), which was computed by dividing the read count at each nt position by the total number of mapped reads and then multiplying the result with 10^6^.

Quantitation of Ribo-seq and RNA-seq data was performed by summing the total number of normalized reads mapping to each coding sequence or UTR regions obtained from published studies.^73,74^ These total number of reads per gene was normalized by the gene’s length (in kilobases) to obtain rpkm values. Ribo-seq reads were shifted 15 nt from their 3′ end to align the P-site to the beginning of each gene. Data from 15 nt of either end of the ORFs was eliminated to reduce the effects of initiation and termination on ribosome occupancy. For differential expression analysis by DESeq2,^72^ raw counts were first generated for each gene. The gene expression profiles were compared by running DESeq2 on Rstudio and Padj values were obtained. We used Padj <0.05 for significance cut-off, log_2_FoldChange value >0.8 for upregulated and < −0.8 for the downregulated genes. Volcano plots were generated by using ggplot2 in Rstudio.^75^ Gene ontology analysis was performed by using PANTHER Classification System (http://www.pantherdb.org/)^76,77^ with following parameters: PANTHER version 17.0 Overrepresentation Test, FISHER test with FDR correction, PANTHER GO-Slim Biological Process with Saccharomyces Cerevisiae - REFLIST (6050) as a reference gene list. Gcn4-target mRNAs were obtained from a published ChIP-seq dataset.^52^ From this dataset, the first 250 genes that had >2-fold increase in Rbp3 (RNA polymerase B) occupancy in starved cells with reproducible induction by Gcn4 in other datasets^78–80^ were defined as Gcn4 targets.

Metagene plots were generated by averaging rpm around the start and stop codons normalized by the total number of reads in a given window for each gene (100 nt upstream of the ORF and 300 nt into the ORF for start codon metagene; 300 nt of the ORF and 100 nt downstream of the ORF for stop codon metagene). ORFs that were unidirectionally overlapping with other ORFs, the genes with features smaller than the window size, and the genes without any mapped reads were excluded from the analysis.

Average reads plots of XIP motifs were generated by first creating a list of occurrences of XIP motifs in the yeast transcriptome and then averaging normalized monosome or disome occupancy from a region of interest (50 nt upstream and 50 nt downstream of XIP motif). Normalization was done by dividing the rpm at each position in the region of interest by the average rpm of the gene.

Pause scores were computed by dividing the rpm of a motif by the average rpm in a region of interest (±50 nt of each motif). Pause scores for sites that are smaller than the ±50 nt window were eliminated from the analysis. Average pause scores were generated by averaging the individual pause scores for each tri-amino acid motif. We excluded the motifs that were represented in the genome less than 100 times to reduce noise, which resulted in 6267 motifs that were compared across datasets. Individual pause scores for XIP motifs were visualized in a boxplot to show the distribution and significance of XIP pause scores. The significance of differences in the median of these individual pause scores were computed by independent 2-group Mann Whitney U Test in Rstudio.

#### Dual luciferase reporter experiments

##### 1. Plasmid building

RLuc-P2A-X-P2A-Fluc plasmids (where X represents a variable sequence) were assembled using NEB Builder HiFi DNA Assembly Cloning Kit (New England Biolabs, #E2621S) by combining the original plasmid (p222) digested with HindIII (New England Biolabs, #R3104S) and NotI (New England Biolabs, #R3189S) and the gene fragments and oligos listed in Table S2.

##### 2. Luminescence activity measurement

Yeast strains transformed with the plasmids described above in log phase grown in SD-Ura medium were pelleted down and transferred to SD-Ura-Met to induce plasmid expression for 90 min. For cells treated with indicated H_2_O_2_ concentrations, plasmid expression was induced for 60 min and then H_2_O_2_ was added to the medium and incubated for 30 min under agitation. Pelleted cells were disrupted by glass bead agitation at 4°C in 1x Passive Lysis Buffer provided in the Dual-Luciferase Reporter Assay System (Promega, #E1910). Extracts were clarified by centrifugation, and protein concentration was determined by BCA assay (ThermoFisher, #23225). The luminescence activities of Rluc and Fluc were collected for 5 μg of protein mixed with the respective substrates. For Figures 3C and S2D, luminescence values were obtained in a VictorX (PerkinElmer) plate reader. For Figure S1D (left panel) luminescence values were obtained using a Glo Max (Promega) plate reader. For Figures 2A, S1D (right panel), S1E and S2E luminescence values were obtained using a CLARIOstar Plus (BMG LabTech) plate reader.

#### Northern blotting

The tRNA-Pro coding sequence was ordered as a gBlock (listed in Table S2) and was assembled with a digested YCplac33 backbone using NEBuilder HiFi DNA Assembly Cloning Kit (NEB, #E5520). The tRNA-Pro probe sequence was amplified from this plasmid using the primers in Table S2 and *in-vitro* transcribed by using Digoxigenin-11-UTP included in DIG Northern Starter Kit (Sigma, #12039672910). 25 μg total RNA each from WT and *rad6*Δ cells was resolved on 15% TBE-Urea polyacrylamide gel (Bio-Rad, #3450091). The RNAs were then transferred onto positively charged nylon membrane (Sigma, #11209299001) in 20x SSC buffer for 3 h by using Nytran SuPerCharge turboblotter system (Cytiva, #10416302), following manufacturer’s instructions. The RNA was UV-crosslinked to the membrane by using a VWR UV crosslinker (VWR, #89131-484) at 120,000 μJ per cm^2^. Then 100 ng/mL of the probe diluted in DIG Easy Hyb Granules Working Buffer was hybridized overnight at 42°C with gentle agitation (Sigma, #11796895001). Next day, the membranes were washed with low stringency wash buffer (2X SSC, 0.1% SDS) and then with high stringency wash buffer (1X SSC, 0.1% SDS), twice for 5 min at room temperature for each. The membrane was washed and subjected to Anti-DIG-AP antibody by using DIG Wash and Block Buffer Set (Sigma, #11585762001) and immonological detection of the membrane was conducted by CDP-Star chemiluminescent substrate included in the northern blotting kit.

#### Translation rate assays

The indicated yeast strains in logarithmic phase (grown in SD medium) were back-diluted to OD_600_ 0.1–0.2 in SD-Met medium. At OD_600_ 0.4–0.5, cells were treated with 50 μM of HPG (L-Homopropargylglycine, Sigma, #900893) and collected by centrifugation after 15, 30, 45, and 60 min of incubation at 30°C under agitation. For H_2_O_2_ treatment, cells were incubated with 0.6 mM of H_2_O_2_ for 15 min prior to HPG incubation as above. Pelleted cells were fixed overnight in 70% ethanol at 4°C and the HPG conjugation with Alexa Fluor 488 was done using the Click-iT HPG Alexa Fluor Protein Synthesis Assay (ThermoFisher, #C10428) following manufacturer’s instructions. Alexa Fluor 488 fluorescent signal was measured in the BD FACS Canto flow cytometer using a 488 nm laser. Single-cell population gates, histograms plots, and mean/median calculations were done using FlowJo software (Becton Dickinson).

#### Western blotting

For blot in Figure 3B and 5C–E, yeast cells grown to logarithmic phase (OD~0.5–0.6) were disrupted by glass-bead agitation at 4°C in buffer containing 50 mM Tris-HCl pH 7.5, 150 mM NaCl, 20 mM iodoacetamide, 1X protease inhibitor cocktail set I (Sigma, #539131). Extracts were clarified by centrifugation, and protein concentration was determined by Bradford assay (Bio-Rad, #5000205) prior to western blotting. Proteins were separated by standard 10% or 12.5% SDS-PAGE loaded in Laemmli buffer and transferred to PVDF membrane (ThermoFisher, #88518). Immunoblotting was performed using the following antibodies: anti-K63 ubiquitin (EMD Millipore, #051308), anti-GAPDH (Abcam, #ab9485), anti-eiF2α-Phospho (Cell Signaling, #3398), anti-actin (Cell Signaling, #4967). For the blot in Figure S4E, yeast extracts were prepared from 25 mL of yeast cells grown to logarithmic phase (OD~0.5–0.6) by TCA precipitation. 10 μL samples were loaded on 4–20% Mini-Protean TGX gel (Bio-Rad, #4561096) and transferred to a PVDF membrane (Bio-Rad, #1704156). The proteins were detected using antibodies against eIF2α-Phospho (Abcam, #32157). The antibody against yeast eIF2α was kindly provided by the laboratory of Thomas Dever (NIH/NICHD).

#### *GCN4-lacZ* reporter assays

Expression of *GCN4-lacZ* fusions was measured by assaying β-galactosidase in whole-cell extracts. Yeast cells transformed with GCN4-lacZ plasmids were grown to logarithmic phase (OD~0.4–0.5) and disrupted by glass-bead agitation at 4°C in buffer containing 1x PBS, 40 mM KCl, and 10 mM MgCl_2_. Extracts were clarified by centrifugation, and protein concentration was determined by BCA (ThermoFisher, #23225) or Bradford assays (Bio-Rad, #5000205). 120 μg protein was mixed with substrate containing 15mM ONPG (2-Nitrophenyl-β-D-galactopyranoside, Goldbio, #N27510), 5mM DTT, 1x PBS, 40mM KCl, and 10mM MgCl_2_, and incubated for 30 min at 30°C. Absorbance was read at 420 nm in a Tecan Sunrise plate reader. When noted, cells were treated with 0.6 mM H_2_O_2_ for 2 h or 30 mM 3-amino-triazole (3-AT) for 5 h in SD media without histidine.

### QUANTIFICATION AND STATISTICAL ANALYSIS

Sample sizes and statistical tests used in the paper are described in the figure legends and further details are provided in the methods section. All statistical analysis were performed on GraphPad Prism, RStudio and DESeq2 software. Differences were considered statistically significant at a p value <0.05. For multiple comparison analyses, post hoc tests were used to access statistical difference between specific groups. Metagene and position average plots were generated by Igor Pro 8 (Wavemetrics). Volcano plots and correlation matrix were generated on RStudio. Scatter and blot plots were generated on GraphPad Prism. Histogram plots were generated on Flow Jo software, also used to calculate mean fluorescent values.

## Supplementary Material

1

2

## Figures and Tables

**Table:** KEY RESOURCES TABLE

REAGENT or RESOURCE	SOURCE	IDENTIFIER
Antibodies		
Anti-eIF2α	Custom antibody made in the laboratory of Thomas Dever (NIH)	N/A
Anti-eIF2α-phospho	Abcam	Cat# ab32157; RRID: AB_732117
Anti-K63 ubiquitin	Sigma-Aldrich	Cat# 05-1308; RRID: AB_1587580
Anti-GAPDH	Abcam	Cat# ab9485; RRID: AB_307275
Anti-actin	Cell Signaling	Cat# 4967; RRID: AB_330288
Anti-eIF2α-phospho	Cell Signaling	Cat# 3398
Anti-H3	Abcam	Cat# ab1791; RRID: AB_302613
Bacterial and virus strains		
NEB^®^ 10-beta Competent *E.coli*	NEB	C3019H
Chemicals, peptides, and recombinant proteins		
D-Glucose	BD Difco	215510
Yeast nitrogen base	BD Difco	291940
Amino acid mix without Leu and Trp	Sigma-Aldrich	Y0750
Amino acid mix without Leu, Trp and Ura	Sigma-Aldrich	Y1771
Amino acid mix without Ura	Sigma-Aldrich	Y1501
Amino acid mix without His, Leu, Trp, Ura	Sigma-Aldrich	Y2001
Amino acid mix without His, Leu, Met, Trp, Ura	Sunrise Science Products	1095
Hydrogen peroxide	Sigma-Aldrich	216763
L-leucine	Sigma-Aldrich	L800
Tryptophan	Sigma-Aldrich	T8941
Uracil	Sigma-Aldrich	U0750
L-Histidine	Sigma-Aldrich	H8000
Cycloheximide	Sigma-Aldrich	C7698
RNase I	Ambion	AM2294
Adenylation mix	NEB	E2610
PNK	NEB	M0201L
T4 RNA Ligase 2 Truncated, K227Q	NEB	M0351L
RecJ exonuclease	Biosearch technologies	RJ411250
5′ deadenylase	NEB	M0331S
FastSelect rRNA removal kit	Qiagen	334215
Superscript III Reverse Transcriptase	Invitrogen	18080044
CircLigase ssDNA ligase	Biosearch technologies	CL4115K
Dynabeads MyOne Streptavidin C1	Invitrogen	65001
Phusion DNA Polymerase	ThermoFisher Scientific	F530L
NEB Builder Hifi DNA Assembly Cloning Kit	NEB	E2621S/E5520S
Restriction Enzyme HindIII	NEB	R31045
Restriction Enzyme NotI	NEB	R31895
DIG Northern Starter Kit	Sigma-Aldrich	12039672910
Oligo Clean & Concentrator kit	Zymo Research	D4060
Quick Start^™^ Bradford 1x Dye Reagent	Bio-Rad	5000205
L-Homopropargylglycine	Sigma-Aldrich	900893
2-Nitrophenyl-β-D-galactopyranoside	Goldbio	N27510
3-amino-1,2,4-triazole	Sigma-Aldrich	A8056
Critical commercial assays		
High Sensitivity DNA Kit	Agilent	5067-4626
High Sensitivity D100 Screen Tape System	Agilent	5067-5584/5585
BCA Assay	ThermoFisher Scientific	23225
Click-iT^®^ HPG Alexa Fluor^®^ Protein Synthesis Assay Kit	ThermoFisher Scientific	C10428
Dual Luciferase^®^ Reporter Assay System	Promega	E1910
Deposited data		
Raw and processed data, see also Table S3	This paper	GEO: GSE226082
Raw western blot gel images	This paper, Mendeley data	https://doi.org/10.17632/j99gggz7ys.1
Experimental models: Organisms/strains		
For yeast strains, see Table S1	N/A	N/A
Oligonucleotides		
See Table S2	This paper	N/A
Recombinant DNA		
See Table S2	N/A	N/A
Software and algorithms		
Scripts used for the analysis	This paper	https://github.com/guydoshlab/Yeastcode1
Igor Pro 8	Wavemetrics	15–500
BOWTIE 1.1.2	Github	Langmead et al.^70^
Cutadapt		Martin^71^
Biopython	Github	https://github.com/biopython/biopython
BCbio	Github	https://github.com/chapmanb/bcbio.variation
DESEQ2	Bioconductor	Love et al.^72^
Rstudio Version 1.3.1093	Rstudio, Inc., Boston, MA	N/A
Prism 9 for macOS, version 9.0.2	GraphPad Software, Inc. San Diego, California USA	www.graphpad.com
FlowJo Version 10.8.1	Becton Dickinson	www.flowjo.com
Other		
15% TBE-Urea polyacrylamide gel	Bio-Rad	3450091
10% TBE-Urea gel	Bio-Rad	3450089
4-20% Mini-Protean TGX gel	Bio-Rad	4561096
PVDF membrane pack	Bio-Rad	1704156
PVDF Transfer Membrane	ThemoFisher Scientific	88518
Nylone Membrane	Sigma-Aldrich	11209299001
Protease Inhibitor Cocktail Set	Sigma-Aldrich	539131

## Highlights

- Rad6 is required for sequence-specific ribosome pausing under oxidative stress
- Rad6 affects translation independently of the ribosome-associated quality-control pathway
- Cells lacking Rad6 show dysregulated translational repression upon oxidative stress
- Loss of Rad6 leads to altered activation of the integrated stress response pathway
