# Supplementary material for: The ubiquitin conjugase Rad6 mediates ribosome pausing during oxidative stress
Source: Cell Rep. Author manuscript; Available in PMC 2023 Dec 29. (PMC10755677; doi:10.1016/j.celrep.2023.113359)
Supplement: 1 [file NIHMS1948193-supplement-1.pdf]

Cell Reports, Volume *42*

Supplemental information

The ubiquitin conjugase Rad6 mediates ribosome pausing during oxidative stress

Sezen Meydan, Géssica C. Barros, Vanessa Simões, Lana Harley, Blanche K. Cizubu, Nicholas R. Guydosh, and Gustavo M. Silva

Figure S1

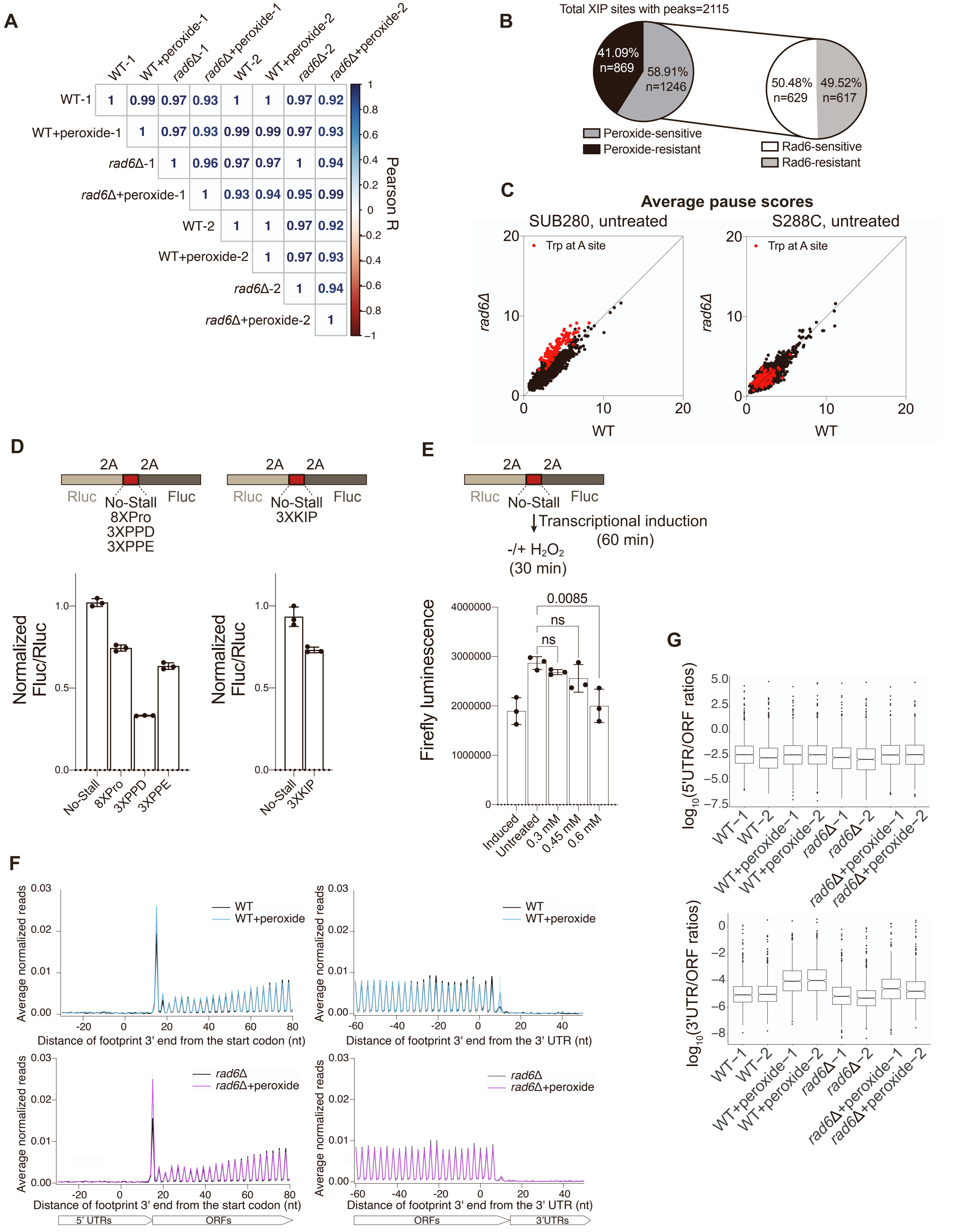

A
WT-1
WT+peroxide-1
rad6Δ-1
rad6Δ+peroxide-1
WT-2
WT+peroxide-2
rad6Δ-2
rad6Δ+peroxide-2
Pearson R
B
Total XIP sites with peaks=2115
41.09% n=869
58.91% n=1246
50.48% n=629
49.52% n=617
Peroxide-sensitive
Peroxide-resistant
Rad6-sensitive
Rad6-resistant
C
Average pause scores
SUB280, untreated
S288C, untreated
Trp at A site
rad6Δ
WT
D
2A
Rluc
Fluc
No-Stall
8XPro
3XPPD
3XPPE
3XKIP
Normalized Fluc/Rluc
E
Transcriptional induction (60 min)
-/+ H2O2 (30 min)
Firefly luminescence
0.0085
ns
Induced
Untreated
0.3 mM
0.45 mM
0.6 mM
F
Average normalized reads
WT
WT+peroxide
rad6Δ
rad6Δ+peroxide
Distance of footprint 3' end from the start codon (nt)
Distance of footprint 3' end from the 3' UTR (nt)
5' UTRs
ORFs
3'UTRs
G
log10(5'UTR/ORF ratios)
log10(3'UTR/ORF ratios)

Figure S1. Global analysis of translation in WT and *rad6*Δ cells. Related to Figures 1 and 2.

(A) Correlation matrix of all RNA-seq data from WT and *rad6*Δ ± H_2O_2 (peroxide) cells, computed using raw counts for each gene. This shows the reproducibility of replicates and extent of change between different conditions. The color scale (right) corresponds to the color of the text within each square.

(B) Pie charts showing the sensitivity of XIP pausing to peroxide and the presence of Rad6. For this analysis, Ribo-seq pause scores corresponding to n=9221 XIP sites in the genome were evaluated. Among these, 2115 XIP sites have >0 reads in all 8 samples (WT, WT+peroxide, *rad6*Δ and *rad6*Δ+peroxide in 2 replicates). Within this group, the XIP sites were considered as "peroxide-sensitive" if the pause score at the site was increased by >2 fold (n=1246) upon peroxide in WT cells and the remaining sites (n=869) were considered as "peroxide-resistant". Within the peroxide-sensitive XIP sites, those that have pause scores decreased >2 fold in *rad6*Δ cells were evaluated as "Rad6-sensitive" (n=629) and the remainder of the sites were grouped as "Rad6-resistant" (n=617).

(C) Average pause scores of 6267 tri-amino acid motifs plotted for untreated WT and *rad6*Δ cells in either SUB280 (left) or S288C (right) background. Motifs with Trp at the A-site are indicated in red. Note that Trp stalling in *rad6*Δ cells is specific to the SUB280 strain.

(D) Schematic of reporter experiments that test motifs with high pause scores in Ribo-seq data (top). As expected, Fluc/Rluc values for stall-inducing sequences, relative to the reporter without any stall sequence (No-Stall), show that stalling motifs cause 20-70% reduction in Fluc and therefore confirm ribosome rescue or drop-off prior to the ribosome

reaching Fluc (bottom). Data from 3 replicates are shown. Error bar indicates mean ± standard deviation. Data are for WT cells.

(E) Schematic of reporter experiments in the presence of peroxide (top). Unlike luciferase assays elsewhere, this assay includes treatment with peroxide, which takes place for 30 min once transcription of the reporter is induced for 60 min. The data (bottom) show firefly luminescence (only) in untreated cells and cells treated with 0.3, 0.45 and 0.6 mM of peroxide. This value increases during the induction phase (induced vs untreated bars) as the cells move toward a steady state. In peroxide-treated cells, the luminescence decreases and is most severe at 0.6 mM (red). This peroxide-dependent baseline makes it difficult to assay additional effects due to stall-inducing sequences. Data from 3 replicates are shown. Error bar indicates mean ± standard deviation. Data are for WT cells.

(F) Metagene analysis showing the average normalized Ribo-seq reads mapped to genes that were aligned by their respective start codon (left panels) or stop codon (right panels) of WT (top) and *rad6*Δ (bottom) cells ± peroxide. Average of two replicates ± standard deviation (shaded) is plotted. Loss of Rad6 does not change the overall translation trends.

(G) Box plots showing the distribution of 5'UTR/ORF (top) or 3'UTR/ORF (bottom) ratios for WT and *rad6*Δ cells ± peroxide. 5'UTR and 3'UTR translation slightly increases with peroxide treatment, and these trends are similar in *rad6*Δ cells. The boxes represent the interquartile range (IQR), and the horizontal line indicates the median. Whiskers show 1.5*IQR and notches indicate 1.58*IQR /√n.

Figure S2

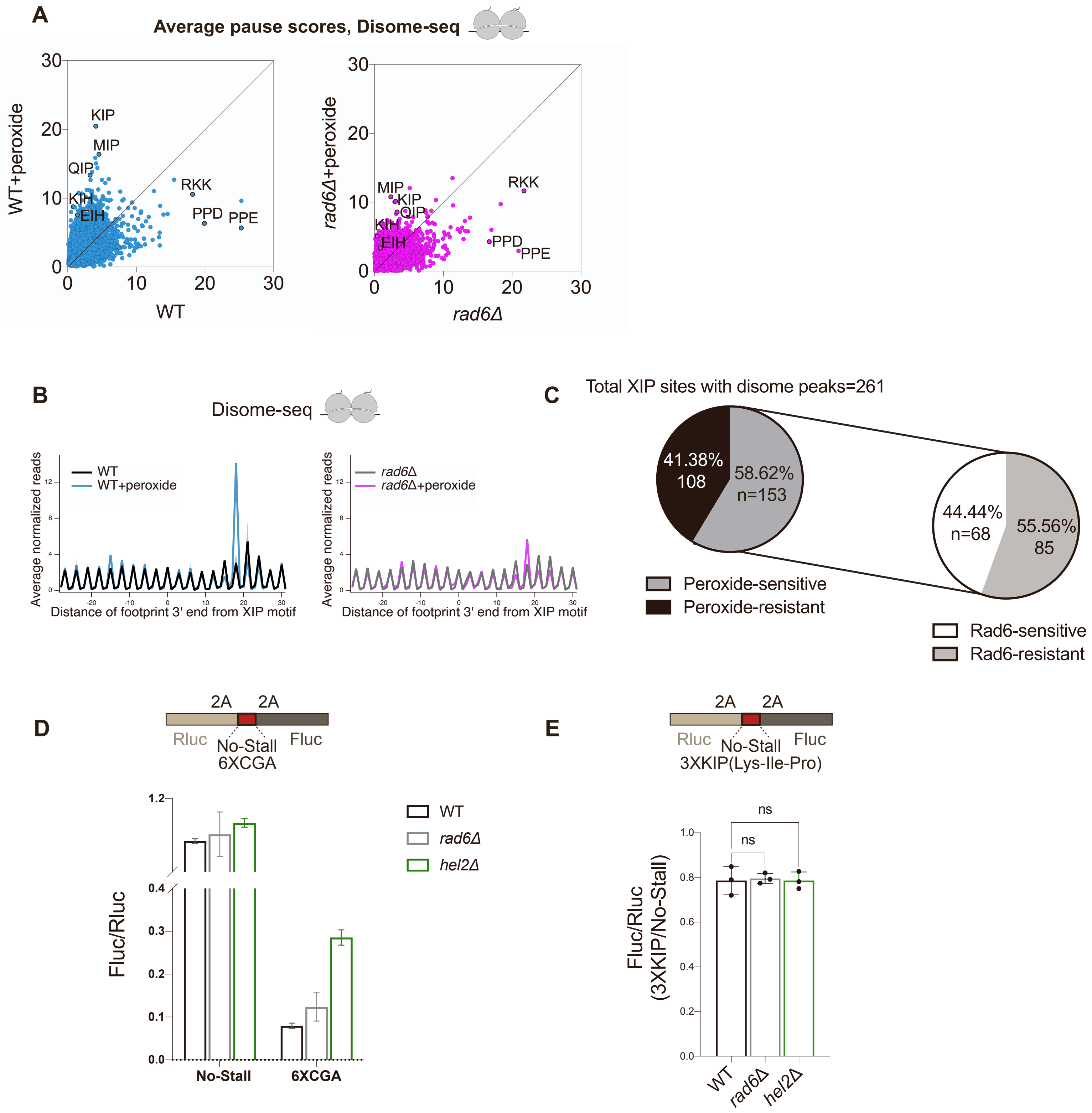

A
Average pause scores, Disome-seq
WT+peroxide
WT
KIP
MIP
QIP
KIH
EIH
RKK
PPD
PPE
rad6Δ+peroxide
rad6Δ
0
10
20
30
B
Disome-seq
Average normalized reads
WT
WT+peroxide
rad6Δ
rad6Δ+peroxide
Distance of footprint 3' end from XIP motif
C
Total XIP sites with disome peaks=261
41.38%
108
58.62%
n=153
44.44%
n=68
55.56%
85
Peroxide-sensitive
Peroxide-resistant
Rad6-sensitive
Rad6-resistant
D
2A 2A
Rluc
No-Stall
6XCGA
Fluc
Fluc/Rluc
1.2
0.4
0.3
0.2
0.1
0.0
No-Stall
6XCGA
WT
rad6Δ
hel2Δ
E
2A 2A
Rluc
No-Stall
3XKIP(Lys-Ile-Pro)
Fluc
Fluc/Rluc
(3XKIP/No-Stall)
ns
ns
1.0
0.8
0.6
0.4
0.2
0.0
WT
rad6Δ
hel2Δ

Figure S2. Disome-seq signatures of WT and *rad6*Δ cells and detailed RQC reporter results. Related to Figures 1 and 3.

(A) Average Disome-seq pause scores of 6267 tri-amino acid motifs plotted for WT and *rad6*Δ cells ± peroxide. The redox pausing signatures are similar to Ribo-seq motifs. Average of two replicates is plotted. Motifs with Trp codons are excluded from this graph.

(B) Average normalized Disome-seq reads mapped to genes aligned by their respective XIP motifs in WT (left) or *rad6*Δ (right) cells. Note that the XIP pausing by disomes is diminished in *rad6*Δ cells, consistent with results for Ribo-seq experiments. Average of two replicates ± standard deviation (shaded) is plotted for WT data and a single replicate is shown for *rad6*Δ cells.

(C) Pie charts showing the sensitivity of XIP disome pausing to peroxide and the presence of Rad6. For this analysis, Disome-seq pause scores corresponding to n=9221 XIP sites in the genome were evaluated. Among these, 261 XIP sites have >0 reads in all 6 samples (WT, WT+peroxide in duplicates, *rad6*Δ and *rad6*Δ+peroxide). Within this group, the XIP sites were considered as "peroxide-sensitive" if the pause score at the site was increased by >2 fold (n=153) upon peroxide in WT cells and the remaining sites (n=108) were considered as "peroxide-resistant". Within the peroxide-sensitive XIP sites, those that have pause scores decreased >2 fold in *rad6*Δ cells were evaluated as "Rad6-sensitive" (n=68) and the remainder of the sites were grouped as "Rad6-resistant" (n=85).

(D) Fluc/Rluc ratio of the dual luciferase reporter with 6xCGA shows that deletion of *HEL2* increases bypassing of the 6xCGA sequence, as anticipated. Data from 3 replicates are shown. Error bar indicates mean ± standard deviation. Loss of the *RAD6* gene, in contrast, does not affect this reporter in a major way.

(E) Schematic for the Renilla-Firefly reporter construct used to measure ribosome rescue at a redox pausing motif (top, 3xKIP). The Fluc/Rluc ratio is expected to become lower when ribosomes dissociate from the mRNA (i.e. via ribosome rescue) after translating the Rluc sequence but prior to reaching the Fluc sequence. The ratio of the Fluc/Rluc value for the 3XKIP reporter compared to a No-Stall reporter is shown (bottom). Deletion of *RAD6* or *HEL2* does not appear to affect ribosome rescue. The significance is assessed by one-way Anova test. ns = not significant. Data from 3 replicates are shown. Error bar indicates mean ± standard deviation.

Figure S3

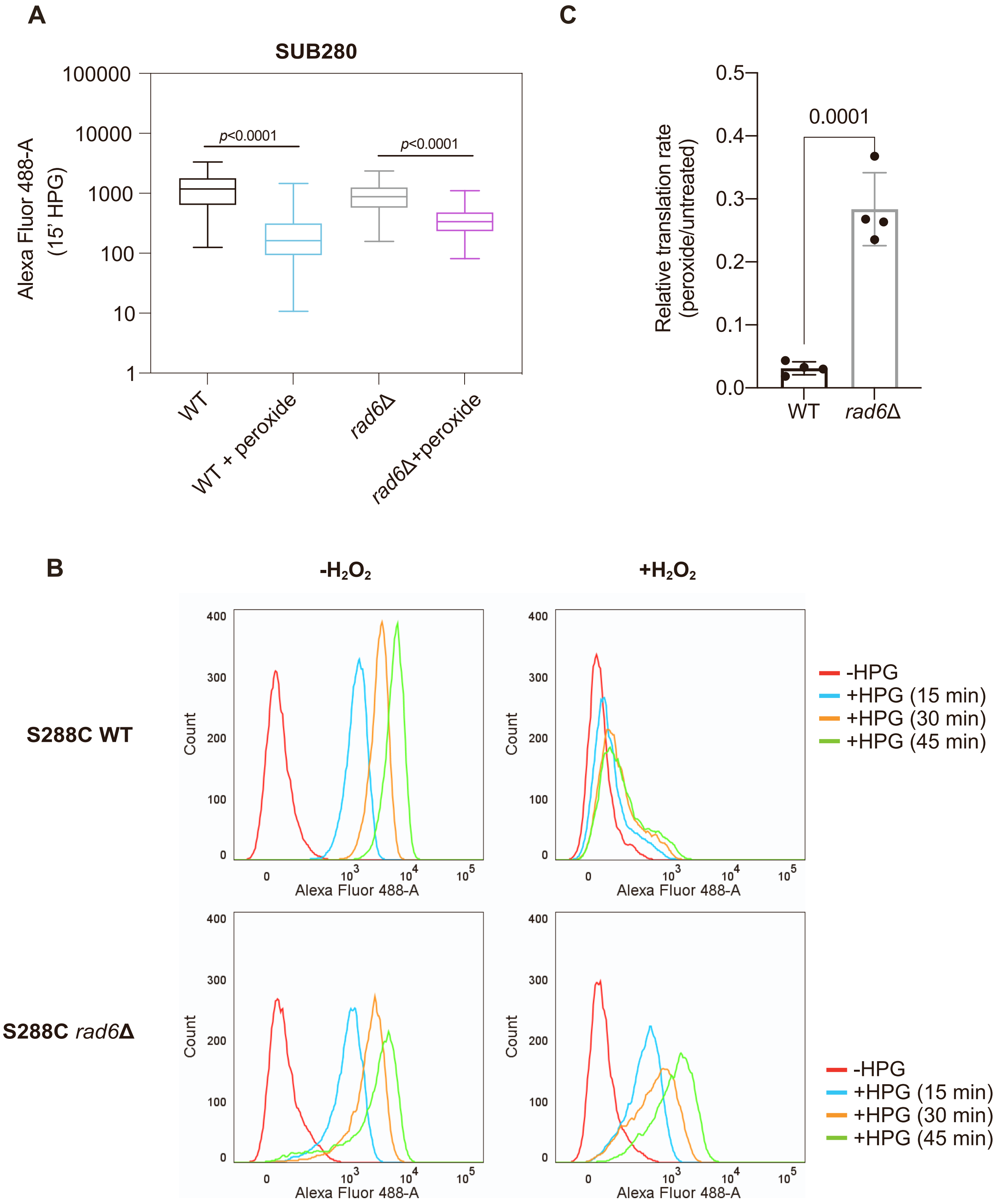

A
SUB280
Alexa Fluor 488-A (15' HPG)
100000
10000
1000
100
10
1
p<0.0001
p<0.0001
WT
WT + peroxide
rad6Δ
rad6Δ+peroxide
C
Relative translation rate (peroxide/untreated)
0.5
0.4
0.3
0.2
0.1
0.0
0.0001
WT
rad6Δ
B
-H2O2
+H2O2
S288C WT
S288C rad6Δ
Count
Alexa Fluor 488-A
-HPG
+HPG (15 min)
+HPG (30 min)
+HPG (45 min)

Figure S3. Translation rate assays in WT and *rad6*Δ cells. Related to Figure 4.

(A) Raw fluorescence data for HPG incorporation during 15 minutes show that peroxide-induced translation inhibition is lower in SUB280 *rad6*Δ cells compared to WT. The statistical significance is assessed by comparing the means via two-way ANOVA test. The line in the middle of each box is plotted at the median and whiskers show the minimum and maximum values.

(B) Histograms generated from HPG incorporation assay showing the number of cells (y-axis) and fluorescence measurements (x-axis) at indicated time points of HPG incubation (15-45 minutes). S288C WT cells exhibit decreased HPG incorporation in the presence of peroxide (top panel). In contrast, HPG incorporation in *rad6*Δ cells is affected less by the peroxide treatment (bottom panel). These data show the responses observed in the SUB280 strain are consistent in the S288C strain.

(C) Quantification of HPG incorporation during peroxide treatment is shown as a normalized rate for HPG incorporation in treated vs untreated S288C cells. The translation rates were calculated by fitting the mean fluorescence values to a linear regression as a function of time. Significance is determined by two-tailed unpaired t-test. Data from 4 replicates are shown. Error bar indicates mean ± standard deviation. These data show the responses observed in the SUB280 strain are consistent in the S288C strain.

Figure S4

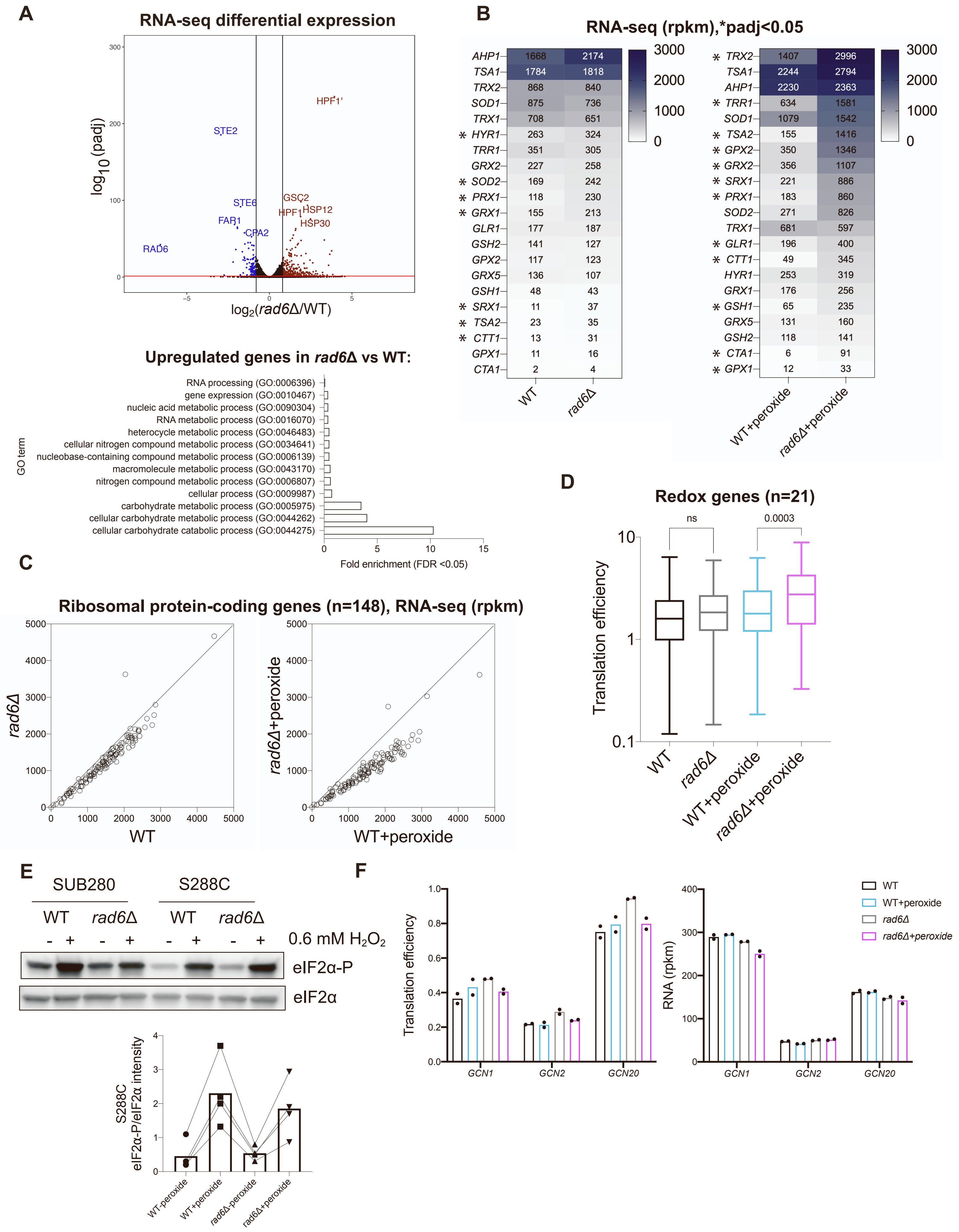
A
RNA-seq differential expression
log10(padj)
log2(rad6Δ/WT)
HPF1'
STE2
STE6
GSC2
HPF1
HSP12
HSP30
FAR1
CPA2
RAD6
Upregulated genes in rad6Δ vs WT:
RNA processing (GO:0006396)
gene expression (GO:0010467)
nucleic acid metabolic process (GO:0090304)
RNA metabolic process (GO:0016070)
heterocycle metabolic process (GO:0046483)
cellular nitrogen compound metabolic process (GO:0034641)
nucleobase-containing compound metabolic process (GO:0006139)
macromolecule metabolic process (GO:0043170)
nitrogen compound metabolic process (GO:0006807)
cellular process (GO:0009987)
carbohydrate metabolic process (GO:0005975)
cellular carbohydrate metabolic process (GO:0044262)
cellular carbohydrate catabolic process (GO:0044275)
GO term
Fold enrichment (FDR <0.05)
B
RNA-seq (rpkm),*padj<0.05
AHP1 1668 2174
TSA1 1784 1818
TRX2 868 840
SOD1 875 736
TRX1 708 651
* HYR1 263 324
TRR1 351 305
GRX2 227 258
* SOD2 169 242
* PRX1 118 230
* GRX1 155 213
GLR1 177 187
GSH2 141 127
GPX2 117 123
GRX5 136 107
GSH1 48 43
* SRX1 11 37
* TSA2 23 35
* CTT1 13 31
GPX1 11 16
CTA1 2 4
WT
rad6Δ
* TRX2 1407 2996
TSA1 2244 2794
AHP1 2230 2363
* TRR1 634 1581
SOD1 1079 1542
* TSA2 155 1416
* GPX2 350 1346
* GRX2 356 1107
* SRX1 221 886
* PRX1 183 860
SOD2 271 826
TRX1 681 597
* GLR1 196 400
* CTT1 49 345
HYR1 253 319
GRX1 176 256
* GSH1 65 235
GRX5 131 160
GSH2 118 141
* CTA1 6 91
* GPX1 12 33
WT+peroxide
rad6Δ+peroxide
C
Ribosomal protein-coding genes (n=148), RNA-seq (rpkm)
rad6Δ
WT
rad6Δ+peroxide
WT+peroxide
D
Redox genes (n=21)
ns
0.0003
Translation efficiency
WT
rad6Δ
WT+peroxide
rad6Δ+peroxide
E
SUB280
S288C
WT
rad6Δ
0.6 mM H2O2
eIF2α-P
eIF2α
S288C eIF2α-P/eIF2α intensity
WT-peroxide
WT+peroxide
rad6Δ-peroxide
rad6Δ+peroxide
F
Translation efficiency
RNA (rpkm)
GCN1
GCN2
GCN20
WT
WT+peroxide
rad6Δ
rad6Δ+peroxide

Figure S4. Differential expression analysis of WT and *rad6*Δ cells. Related to Figure 5.

(A) Volcano plot showing the differential RNA expression in *rad6*Δ versus WT cells. Genes that are significantly upregulated (log_2Fold Change > 0.8, padj < 0.05) or downregulated (log_2Fold Change < -0.8, padj < 0.05), as determined by DESeq2 analysis are shown in red and blue, respectively. The significance cut-off is indicated with a red bar. Significantly enriched gene ontology (GO) terms of the genes that are significantly upregulated in the absence of Rad6 are shown at the bottom. GO analysis is conducted in PANTHER, GO-Slim Biological Process by using *Saccharomyces cerevisiae* (all genes in database) as reference list and test type as FISHER with FDR correction. Genes that are significantly downregulated in *rad6*Δ cells did not have a significantly enriched GO term.

(B) Heatmap showing the RNA-seq expression (rpkm) of redox genes in the WT and *rad6*Δ ± peroxide samples. The plotted data shows the average of two RNA-seq replicates. The genes that are differentially expressed (DESeq2, padj < 0.05) are indicated with an asterisk (*).

(C) RNA-seq data of ribosomal protein-encoding genes (n=148, gene names obtained from SGD) in WT vs *rad6*Δ cells ± peroxide show that mRNAs that encode ribosomal proteins are lower in *rad6*Δ cells. Average of 2 replicates is shown.

(D) Translation efficiency (TE, Ribo-seq reads normalized by RNA-seq reads) of 21 mRNAs that encode redox enzymes shows translational upregulation of these genes in *rad6*Δ vs WT cells in the presence of peroxide. Significance is calculated by one-way ANOVA test. ns = not significant. Average data from 2 replicates were used to compute

TE values. The line in the middle of each box is plotted at the median for the 21 genes and whiskers show the minimum and maximum values.

(E) Western blotting demonstrates that the phosphorylation of eIF2α due to peroxide is minimal in *rad6*Δ cells in the SUB280 background but is more moderate in the S288C background. Bar chart of means below blot shows quantified results for 4 replicates of S288C data (including the above blot) where loss of *RAD6* consistently results in loss of phosphorylation. This suggests that other inputs regulate eIF2α phosphorylation in the S288C background. Note that total eIF2α levels do not change with peroxide and in the different strains.

(F) Bar graphs of mean translation efficiency (left) and RNA levels (right) of GCN1, GCN2, GCN20 genes show that the abundance and translation level of the mRNAs encoding these proteins are not affected by loss of Rad6.

Figure S5

A

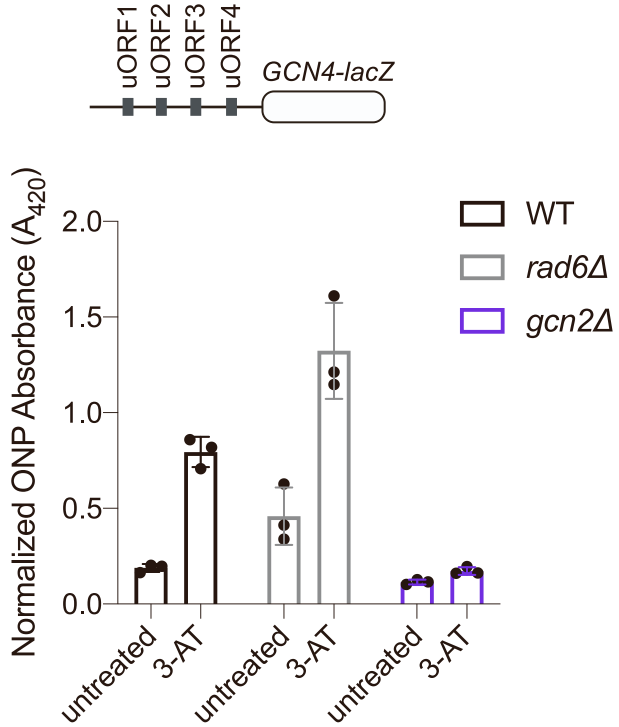

C

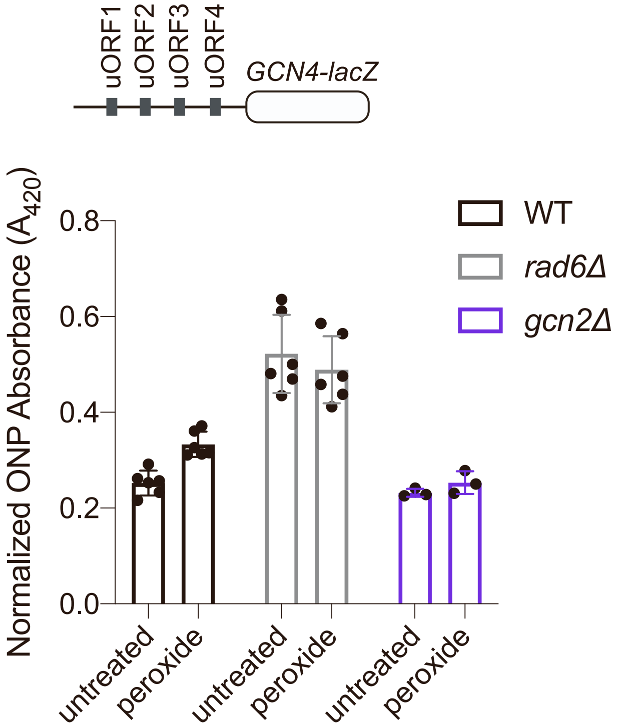

B **Genes coding for initiation factors n=31**

ANB1
CDC33
DED1
FUN12
GCD1
GCD10
GCD11
GCD14
GCD2
GCD6
GCD7
GCN3
HCR1
HYP2
NIP1
NOP6
PRT1
RPG1
SUI1
SUI2
SUI3
TIF1
TIF11
TIF2
TIF3
TIF34
TIF35
TIF4631
TIF4632
TIF5
TIF6

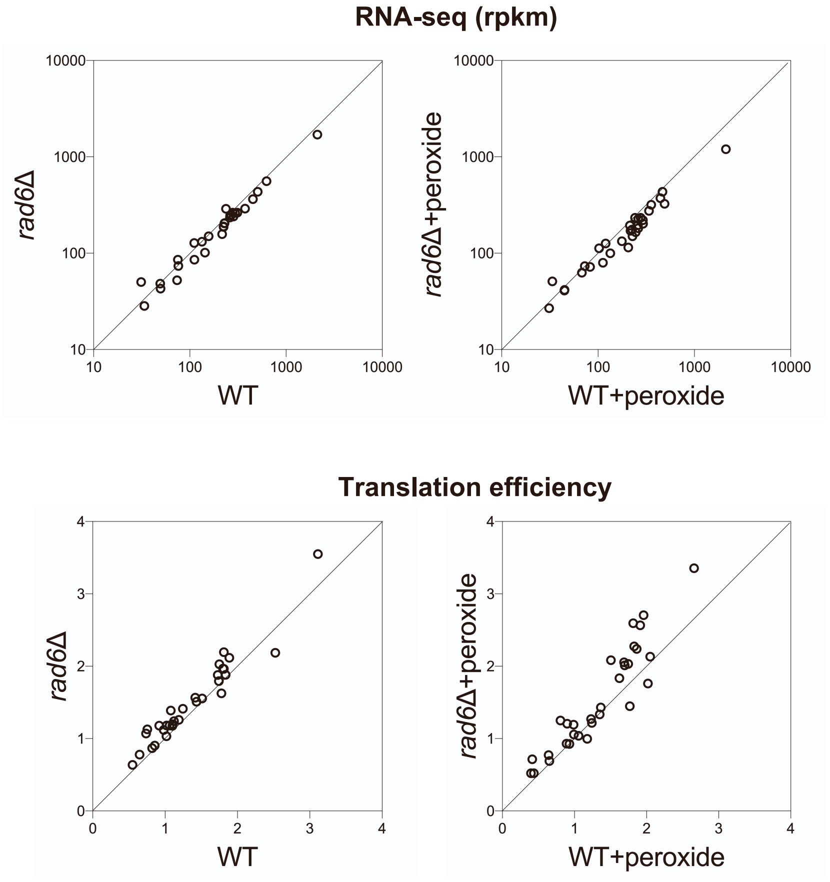

Figure S5. Supporting *GCN4-lacZ* reporter experiments and analysis of expression of *GCD* genes. Related to Figure 6.

(A) *GCN4-lacZ* assay in the presence of 3-AT shows that *GCN4* translation is induced with 3-AT treatment, and this is dependent on the presence of Gcn2. These positive controls show the proper functioning of the reporter in these cells and also show that Gcn2 remains active in *rad6*Δ cells. Data from 3 replicates shown. Error bar indicates mean ± standard deviation.

(B) RNA-seq (top) and translation efficiency (bottom) data corresponding to mRNAs that encode "Gcd" genes indicate that the expression of these genes does not change in the cells lacking Rad6 ± peroxide. The average of 2 replicates is shown.

(C) *GCN4-lacZ* assay performed in the presence of peroxide. The data show that *GCN4* translation is higher in *rad6*Δ cells compared to WT, with and without peroxide.

Data from 3 replicates shown. Error bar indicates mean ± standard deviation.

Table S1. Yeast strains used in this study.

Yeast name	Genotype	Source
SUB280 WT	*MATa lys2-801 leu2-3,112 ura3-52 his3- Δ200 trp1-1[am] ubi1-Δ1::TRP1 ubi2-Δ2::ura3 ubi3-Δub-2 ubi4-Δ2::LEU2 [pUB39 Ub, LYS2][pUB100 Ubi1 tail, HIS3]*	[S1]
SUB280 *rad6*Δ	*MATa lys2-801 leu2-3,112 ura3-52 his3- Δ200 trp1-1[am] ubi1-Δ1::TRP1 ubi2-Δ2::ura3 ubi3-Δub-2 ubi4-Δ2::LEU2 [pUB39 Ub, LYS2][pUB100 Ubi1 tail, HIS3] rad6:kanMX4*	[S2]
SUB280 *hel2*Δ	*MATa lys2-801 leu2-3,112 ura3-52 his3- Δ200 trp1-1[am] ubi1-Δ1::TRP1 ubi2-Δ2::ura3 ubi3-Δub-2 ubi4-Δ2::LEU2 [pUB39 Ub, LYS2][pUB100 Ubi1 tail, HIS3] hel2::kanMX4*	[S3]
SUB280 *rad6*Δ *RAD6*	*MATa lys2-801 leu2-3,112 ura3-52 his3- Δ200 trp1-1[am] ubi1-Δ1::TRP1 ubi2-Δ2::ura3 ubi3-Δub-2 ubi4-Δ2::LEU2 [pUB39 Ub, LYS2][pUB100 Ubi1 tail, HIS3] [pYES RAD6-HA, URA3]*	[S4]
SUB280 *rad6*Δ *RAD6 (C88A)*	*MATa lys2-801 leu2-3,112 ura3-52 his3- Δ200 trp1-1[am] ubi1-Δ1::TRP1 ubi2-Δ2::ura3 ubi3-Δub-2 ubi4-Δ2::LEU2 [pUB39 Ub, LYS2][pUB100 Ubi1 tail, HIS3] [pYES RAD6(C88A)-HA, URA3]*	[S4]
S288C WT	*MATa SUC2 gal2 mal2 mel flo1 flo8-1 hap1 ho bio1 bio6*	[S5]
S288C *rad6*Δ	*MATa SUC2 gal2 mal2 mel flo1 flo8-1 hap1 ho bio1 bio6 rad6::kanMX4*	[S6]

Table S2. Oligonucleotides and plasmids used in this study.

**Name**	**Sequence (5' to 3')**
**Gene fragments and oligo used for generating p416-Met25-Rluc-P2A-X-P2A Fluc plasmids**	
**No-Stall (dsDNA)**	AGAAGATGCACCTGATGAAATGGGAAAATATATCAAATCGTTCGTTGAGCGA<br>GTTCTCAAAAATGAACAAGGAAGCGGAGCTACTAACTTCAGCCTGCTGAAGC<br>AGGCTGGAGACGTGGAGGAGAACCCTGGACCTAAGCTT**GAATTCGATATC**G<br>CGGCCGCTGGAAGCGGAGCAACCAATTTCTCACTATTAAAACAAGCAGGCGA<br>TGTTGAAGAAAATCCAGGTCCGATGGAAGATGCTAAGAATATTAAGAAAGGA<br>CCAGCTCCTTTCTACCCTCTCGAAGATGGAACTGCTGGTGA
**6XCGA (dsDNA)**	AGATGCACCTGATGAAATGGGAAAATATATCAAATCGTTCGTTGAGCGAGTTC<br>TCAAAAATGAACAAGGAAGCGGAGCTACTAACTTCAGCCTGCTGAAGCAGGC<br>TGGAGACGTGGAGGAGAACCCTGGACCTAAGCTT**CGACGACGACGACGAC**<br>**GA**GCGGCCGCTGGAAGCGGAGCAACCAATTTCTCACTATTAAAACAAGCAGG<br>CGATGTTGAAGAAAATCCAGGTCCGATGGAAGATGCTAAGAATATTAAGAAA<br>GGACCAGCTCCTTTCTACCCTCTCGAAGATGGAACTGCTGG
**8xPro (dsDNA)**	TGCACCTGATGAAATGGGAAAATATATCAAATCGTTCGTTGAGCGAGTTCTCA<br>AAAATGAACAAGGAAGCGGAGCTACTAACTTCAGCCTGCTGAAGCAGGCTGG<br>AGACGTGGAGGAGAACCCTGGACCTAAGCTT**CCACCCCCGCCTCCACCCCC**<br>**GCCT**GCGGCCGCTGGAAGCGGAGCAACCAATTTCTCACTATTAAAACAAGCA<br>GGCGATGTTGAAGAAAATCCAGGTCCGATGGAAGATGCTAAGAATATTAAGA<br>AAGGACCAGCTCCTTTCTACCCTCTCGAAGATGGAACTGC
**3xPPD (dsDNA)**	GCACCTGATGAAATGGGAAAATATATCAAATCGTTCGTTGAGCGAGTTCTCAA<br>AAATGAACAAGGAAGCGGAGCTACTAACTTCAGCCTGCTGAAGCAGGCTGGA<br>GACGTGGAGGAGAACCCTGGACCTAAGCTT**CCACCTGATCCACCTGATCCA**<br>**CCTGAT**GCGGCCGCTGGAAGCGGAGCAACCAATTTCTCACTATTAAAACAAG<br>CAGGCGATGTTGAAGAAAATCCAGGTCCGATGGAAGATGCTAAGAATATTAA<br>GAAAGGACCAGCTCCTTTCTACCCTCTCGAAGATGGAACT
**3xPPE (dsDNA)**	GCACCTGATGAAATGGGAAAATATATCAAATCGTTCGTTGAGCGAGTTCTCAA<br>AAATGAACAAGGAAGCGGAGCTACTAACTTCAGCCTGCTGAAGCAGGCTGGA<br>GACGTGGAGGAGAACCCTGGACCTAAGCTT**CCTCCAGAACCTCCAGAACCT**<br>**CCAGAA**GCGGCCGCTGGAAGCGGAGCAACCAATTTCTCACTATTAAAACAAG<br>CAGGCGATGTTGAAGAAAATCCAGGTCCGATGGAAGATGCTAAGAATATTAA<br>GAAAGGACCAGCTCCTTTCTACCCTCTCGAAGATGGAACT
**3xKIP (ssDNA Oligo)**	AGACGTGGAGGAGAACCCTGGACCTAAGCTT**AAAATTCCGAAAATTCCGAA**<br>**AATTCCG**GCGGCCGCTGGAAGCGGAGCTACTAACTTCAGCC
**Oligos used for generating northern blot probe**	
**tRNA-Pro-AGG_Fwd**	GGGCGTGTGGTCTAGAGGTATG
**T7_tRNA-Pro-AGG_Rev**	TAATACGACTCACTATAGGGGAGCCGGGACTCGAACCCGG

**T7_tRNA-Pro-AGG_YCplac33 (gBlock)**	ACCATGATTACGCCAAGCTTGGGCGTGTGGTCTAGAGGTATGATTCTCGCTTAGGGTGCGGGAGGTCCCGGGTTCGAGTCCCGGCTCGCCCCCCTATAGTGAGTCGTATTAGAATTCACTGGCCGTCGTTT
**RNA size selection markers**	
25mer	rArUrGrUrArCrArCrGrGrArGrUrCrGrArGrCrArCrCrCrGrCrA
34mer	rArUrGrUrArCrArCrGrGrArGrUrCrGrArGrCrArCrCrCrGrCrArArCrGrCrGrArArUrG
54mer	rArUrGrUrArCrArCrGrGrArGrUrCrGrArGrCrArCrCrCrGrCrArArCrGrCrGrArArUrGrUrArCrArCrGrGrArGrUrCrGrArGrCrArCrCrCrG
68mer	rArUrGrUrArCrArCrGrGrArGrUrCrGrArGrCrArCrCrCrGrCrArArCrGrCrGrArArUrGrUrArCrArCrGrGrArGrUrCrGrArGrCrArCrCrCrGrCrArArCrGrCrGrArUrGrUrArCrA
70mer	rArUrGrUrArCrArCrGrGrArGrUrCrGrArCrCrCrGrCrArArCrGrCrGrArUrGrUrArCrArCrGrGrArGrUrCrGrArCrCrCrGrCrArArCrGrCrGrArUrGrUrArCrArCrGrGrArGrUrCrGrA
**rRNA substraction oligonucleotides**	
1b	/5BioTinTEG/GGTGCACAATCGACCGATC
2b	/5BioTinTEG/GTTTCTTTACTTATTCAATGAAGCGG
3b	/5BioTinTEG/TATAGATGGATACGAATAAGGCGTC
4	/5BioTinTEG/TTGTGGCGTCGCTGAACCATAG
5	/5BioTinTEG/CAGGGGGCATGCCTGTTTGAGCGTCAT
6	/5BioTinTEG/CGGTGCCCGAGTTGTAATTT
**Linker oligonucleotides**	
NI-810	5´-/5Phos/NNNNNATCGTAGATCGGAAGAGCACACGTCTGAA/3ddC/
NI-811	5´-/5Phos/NNNNNAGCTAAGATCGGAAGAGCACACGTCTGAA/3ddC/
NI-812	5´-/5Phos/NNNNNCGTAAAGATCGGAAGAGCACACGTCTGAA/3ddC/
NI-813	5´-/5Phos/NNNNNCTAGAAGATCGGAAGAGCACACGTCTGAA/3ddC/
NI-814	5´-/5Phos/NNNNNGATCAAGATCGGAAGAGCACACGTCTGAA/3ddC/
NI-815	5´-/5Phos/NNNNNGCATAAGATCGGAAGAGCACACGTCTGAA/3ddC/
**RT primer**	
NI-802	5´-/5Phos/NNAGATCGGAAGAGCGTCGTGTAGGGAAAGAG/iSp18/GTGACTGGAGTTCAGACGTGTGCTC
**PCR primers**	
NI-NI-798	5´-AATGATACGGCGACCACCGAGATCTACACTCTTTCCCTACACGACGCTC
NI-799	5´-CAAGCAGAAGACGGCATACGAGATCGTGATGTGACTGGAGTTCAGACGTGTG
NI-822	5’-CAAGCAGAAGACGGCATACGAGATACATCGGTGACTGGAGTTCAGACGTGTG

NI-823	5'-CAAGCAGAAGACGGCATACGAGATGCCTAAGTGACTGGAGTTCAGACGTGTG		
NI-824	5'-CAAGCAGAAGACGGCATACGAGATTGGTCAGTGACTGGAGTTCAGACGTGTG		
NI-825	5'-CAAGCAGAAGACGGCATACGAGATCACTGTGTGACTGGAGTTCAGACGTGTG		
**Plasmids**			
**Plasmid No.**	**Vector**	**Gene**	**Reference**
**p028**	pYES 2.0	*RAD6*	[S4]
**p032**	pYES 2.0	*RAD6* (C88S)	[S4]
**p116**	pYES 2.0	*RAD6* (C88A)	[S4]
**p149**	p180	*Gcn4-LacZ* (4 uORFs)	[S7]
**p150**	p227	*Gcn4-LacZ* (uORFless)	[S8]
**p222**	p416 (Met25p)	Rluc-P2A-NoStall-P2A-Fluc	This study
**p223**	p416 (Met25p)	Rluc-P2A-3xKIP-P2A-Fluc	This study
**p250**	p416 (Met25p)	Rluc-P2A-6xCGA-P2A-Fluc	This study
**p255**	pM199Z	*Gcn4-LacZ* (uORF1 only)	[S9]
**p256**	pM226Z	*Gcn4-LacZ* (uORF1 extended to *GCN4* main ORF)	[S9]
**p259**	p416 (Met25p)	Rluc-P2A-8xPro-P2A-Fluc	This study
**p261**	p416 (Met25p)	Rluc-P2A-3xPPD-P2A-Fluc	This study
**p262**	p416 (Met25p)	Rluc-P2A-3xPPE-P2A-Fluc	This study

Table S3. Ribosome profiling statistics.

Sample name	Description	Data type	Reads aligned to non-coding RNA	Not aligned to non-coding RNA	Reads without PCR duplicates	Aligned to coding regions and splice junctions
SM061F	SUB280 WT_rep1	Ribo-seq	88,465,576	32,310,998	21,072,905	16,214,578
SM071F	SUB280 WT_rep2	Ribo-seq	81,035,725	41,623,662	29,668,687	24,022,020
SM062F	SUB280 WT+peroxide_rep1	Ribo-seq	106,692,905	28,338,349	18,298,626	13,749,540
SM072F	SUB280 WT+peroxide_rep2	Ribo-seq	78,751,014	26,997,840	19,255,686	15,370,434
SM065F	SUB280 *rad6*Δ_rep1	Ribo-seq	91,450,355	32,530,594	13,938,148	10,430,067
SM075F	SUB280 *rad6*Δ_rep2	Ribo-seq	102,956,589	43,203,482	30,983,491	24,965,084
SM066F	SUB280 *rad6*Δ+peroxide_rep1	Ribo-seq	83,007,104	20,487,638	8,791,438	6,516,848
SM076F	SUB280 *rad6*Δ+peroxide_rep2	Ribo-seq	83,266,747	28,388,419	20,644,308	17,042,575
SM099F	SUB280 *hel2*Δ	Ribo-seq	51,976,975	19,589,386	17,383,785	13,504,129
SM100F	SUB280 *hel2*Δ+peroxide	Ribo-seq	39,728,199	17,108,518	15,205,421	10,719,351
SM103F	SUB280 *rad6*Δ *RAD6*	Ribo-seq	40,183,394	26,152,266	20,545,187	15,387,454
SM104F	SUB280 *rad6*Δ *RAD6*+peroxide	Ribo-seq	38,330,665	16,932,848	13,310,052	10,124,867
SM105F	SUB280 *rad6*Δ *RAD6 (C88A)*	Ribo-seq	73,490,623	39,788,606	30,908,395	24,485,828
SM106F	SUB280 *rad6*Δ *RAD6 (C88A)*+peroxide	Ribo-seq	46,123,764	21,249,347	16,905,721	13,384,694
SM107F	S288C WT	Ribo-seq	43,906,581	18,322,535	15,662,623	11,286,980

SM108F	S288C WT+peroxide	Ribo-seq	46,024,866	10,218,846	8,698,065	6,022,004
SM109F	S288C *rad6*Δ	Ribo-seq	76,335,435	27,695,810	23,610,645	17,854,558
SM110F	S288C *rad6*Δ+peroxide	Ribo-seq	10,886,937	3,519,792	3,057,926	2,286,614
SM061Fd	SUB280 WT_rep1	Disome-seq	7,761,659	5,475,884	3,524,369	964,664
SM071Fd	SUB280 WT_rep2	Disome-seq	51,196,443	30,402,393	5,154,631	2,895,728
SM062Fd	SUB280 WT+peroxide_rep1	Disome-seq	6,670,378	5,794,983	3,619,232	1,318,655
SM072Fd	SUB280 WT+peroxide_rep2	Disome-seq	31,647,008	37,880,232	6,159,970	3,965,524
SM065Fd	SUB280 *rad6*Δ	Disome-seq	6,889,699	7,241,843	2,174,826	1,285,476
SM066Fd	SUB280 *rad6*Δ+peroxide	Disome-seq	4,596,077	7,346,885	2,800,625	733,394
SM061M	SUB280 WT_rep1	RNA-seq	72,941,175	34,744,878	24,913,881	16,298,506
SM071M	SUB280 WT_rep2	RNA-seq	68,143,036	26,850,953	20,027,139	11,889,363
SM062M	SUB280 WT+peroxide_rep1	RNA-seq	61,818,591	26,895,510	19,329,586	12,833,259
SM072M	SUB280 WT+peroxide_rep2	RNA-seq	73,905,111	26,350,153	19,532,817	11,208,925
SM065M	SUB280 *rad6*Δ_rep1	RNA-seq	100,532,895	34,833,857	24,941,388	15,815,962
SM075M	SUB280 *rad6*Δ_rep2	RNA-seq	68,204,722	23,096,946	16,515,446	8,793,155
SM066M	SUB280 *rad6*Δ+peroxide_rep1	RNA-seq	146,467,948	40,302,699	29,535,984	15,823,150
SM076M	SUB280 *rad6*Δ+peroxide_rep2	RNA-seq	81,834,980	20,039,453	14,121,673	7,496,153

BIBLIOGRAPHY

[S1] Finley, D., Sadis, S., Monia, B.P., Boucher, P., Ecker, D.J., Crooke, S.T., and Chau, V. (1994). Inhibition of proteolysis and cell cycle progression in a multiubiquitination-deficient yeast mutant. Mol Cell Biol *14*, 5501-5509. 10.1128/mcb.14.8.5501-5509.1994

[S2] Silva, G.M., Finley, D., and Vogel, C. (2015). K63 polyubiquitination is a new modulator of the oxidative stress response. Nat Struct Mol Biol *22*, 116-123. 10.1038/nsmb.2955

[S3] Back, S., Gorman, A.W., Vogel, C., and Silva, G.M. (2019). Site-Specific K63 Ubiquitinomics Provides Insights into Translation Regulation under Stress. J Proteome Res *18*, 309-318. 10.1021/acs.jproteome.8b00623

[S4] Simoes, V., Cizubu, B.K., Harley, L., Zhou, Y., Pajak, J., Snyder, N.A., Bouvette, J., Borgnia, M.J., Arya, G., Bartesaghi, A., and Silva, G.M. (2022). Redox-sensitive E2 Rad6 controls cellular response to oxidative stress via K63-linked ubiquitination of ribosomes. Cell Rep 39, 110860. 10.1016/j.celrep.2022.110860.

[S5] Mortimer, R.K., and Johnston, J.R. (1986). Genealogy of principal strains of the yeast genetic stock center. Genetics *113*, 35-43. 10.1093/genetics/113.1.35.

[S6] Winzeler, E.A., Shoemaker, D.D., Astromoff, A., Liang, H., Anderson, K., Andre, B., Bangham, R., Benito, R., Boeke, J.D., Bussey, H., et al. (1999). Functional characterization of the S. cerevisiae genome by gene deletion and parallel analysis. Science *285*, 901-906. 10.1126/science.285.5429.901.

[S7] Hinnebusch, A.G. (1985). A hierarchy of trans-acting factors modulates translation of an activator of amino acid biosynthetic genes in Saccharomyces cerevisiae. Mol Cell Biol *5*, 2349-2360. 10.1128/mcb.5.9.2349-2360.1985.

[S8] Mueller, P.P., and Hinnebusch, A.G. (1986). Multiple upstream AUG codons mediate translational control of GCN4. Cell *45*, 201-207. 10.1016/0092-8674(86)90384-3.

[S9] Grant, C.M., Miller, P.F., and Hinnebusch, A.G. (1994). Requirements for intercistronic distance and level of eukaryotic initiation factor 2 activity in reinitiation on GCN4 mRNA vary with the downstream cistron. Mol Cell Biol *14*, 2616-2628. 10.1128/mcb.14.4.2616-2628.1994.
